# Supplementary material for: Effect of the Solvate Environment of Lithium Cations on the Resistance of the Polymer Electrolyte/Electrode Interface in a Solid-State Lithium Battery
Source: Membranes (Basel). 2022 Nov 8;12(11):1111. doi: 10.3390/membranes12111111 (PMC9694555; doi:10.3390/membranes12111111)
Supplement: Supplementary file 1 [file membranes-12-01111-s001.zip › membranes-1971041-supplementary.pdf]

Electronic supplementary information

The effect of the solvate environment of lithium cations on the resistance of polymer electrolyte/electrode interface in solid- state lithium battery

Alexander V. Chernyak, Nikita A. Slesarenko, Anna A. Slesarenko, Guzaliya R. Baymuratova, Galiya Z.Tulibaeva, Alena V.Yudina, Vitaly I. Volkov, Alexander F. Shestakov and Olga V. Yarmolenko

Table of Contents

Table S1. Electrolyte Compositions and Equivalent Cell Circuits $LiFePO_4$//$LiFePO_4$ 3

Table S2. Calculated parameters of equivalent circuits of cells 1-3 4

Figure S1. 1H NMR (a) and 13C NMR (b) high resolution spectrum of GBL 5

Figure S2. 1H NMR (a) and 13C NMR (b) high resolution spectrum of DOL 6

Figure S3. 1H NMR (a) and 13C NMR (b) high resolution spectrum of DME 7

Figure S4. 1H NMR (a) and 13C NMR (b) high resolution spectrum of DOL+DME 8

Assignment of NMR lines of solvent spectra 9

Figure S5. 1H NMR spectrum of electrolytes a) №1 b), №2, c) №3, d) №4. 10

Figure S6. 7Li NMR spectrum of electrolytes a) №1 b), №2, c) №3, d) №4. 11

Figure S7. 11B NMR spectrum of electrolytes a) №1 b), №2, c) №4. 12

Figure S8. 13C NMR spectrum of electrolytes a) №1 b), №2, c) №3, d) №4. 13

Figure S9. 19F NMR spectrum of electrolytes a) №1 b), №2, c) №3, d) №4. 14

Assignment of NMR lines of electrolyte spectra 15

Table S3. Calculated and corrected values of ^{1}H chemical shifts of solvent molecules for different model solvate complexes in ppm 17

Table S4. Energies of formation at T=0 K, dE, and enthalpies of formation dH at T=298 K of solvate complexes 18

Figure S10. Calculated structure of solvate complex $Li^+(DME)_4$ (a), $Li^+(DME)_3$ (b), 19

$Li^+(DOL)(DME)_3$ (c), $Li^+(DOL)_2(DME)_2$ (d), $Li^+(DOL)_3(DME)$ (e), $Li^+(DOL)_4$ (f), $Li^+(GBL)_4$ (g). Bond lengths are given in Å

Table S5. Cartesian coordination of calculated structures in Å 21

Table S1. Electrolyte Compositions and Equivalent Cell Circuits $LiFePO_4//LiFePO_4$

№	Electrolyte	$LiFePO_4$ treatment	Equivalent Circuits*
№1	NPE membrane	no	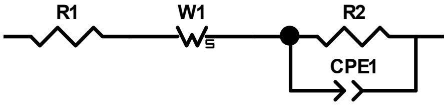
№2	NPE membrane	1MLiBF_4in GBL	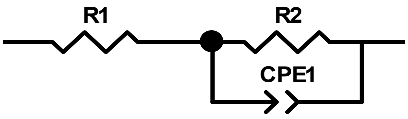
№3	NPE membrane	1 M LiTFSI in DOL/DME (1:1)	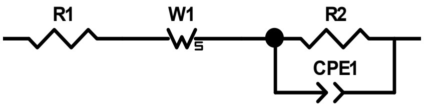

* Elements of equivalent circuits: *R*1 - electrolyte resistance, *R*2 – electrode/electrolyte interface resistance, *CPE*1 – constant phase element, *W*1 – closed Warburg element.

Table S2. Calculated parameters of equivalent circuits of cells 1-3

Cell number	№1	№2	№3
*R*1, Ohm	0.87	0.77	0.27
*R*2, Ohm	5	357	2.08
*CPE*1-*T, F*	2.3×10^{-5}	4.8×10^{-5}	4.5×10^{-3}
*CPE*1-*P*	0.92	0.61	0.9
*W*1-*R,* Ohm	5.1	-	7.2
*W*1-*T,* sec	0.00003	-	0.000026
*W*1-*P*	0.37	-	0.34

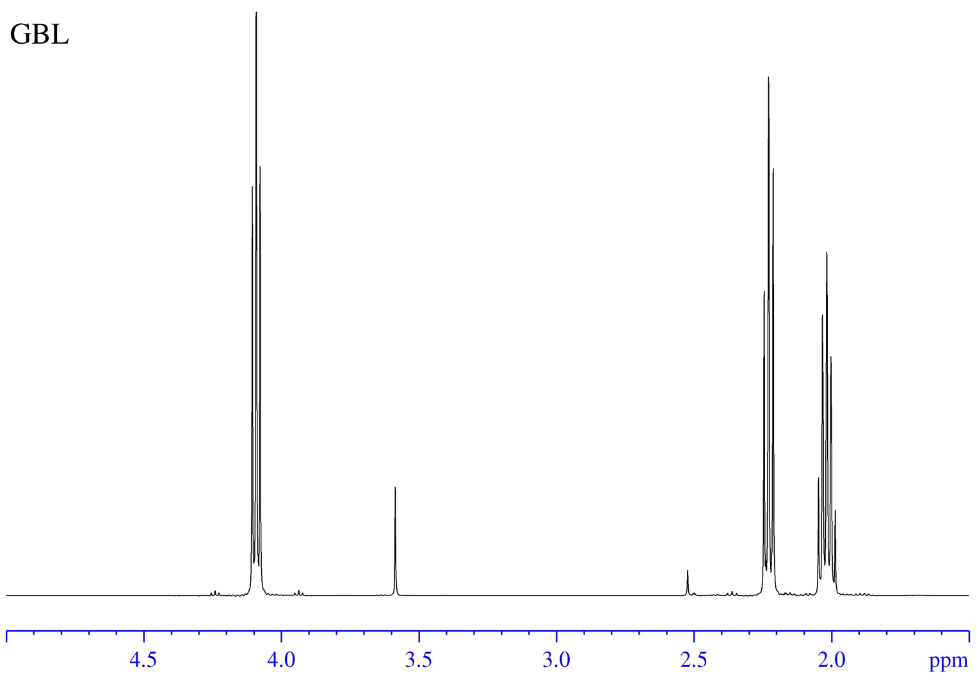

a

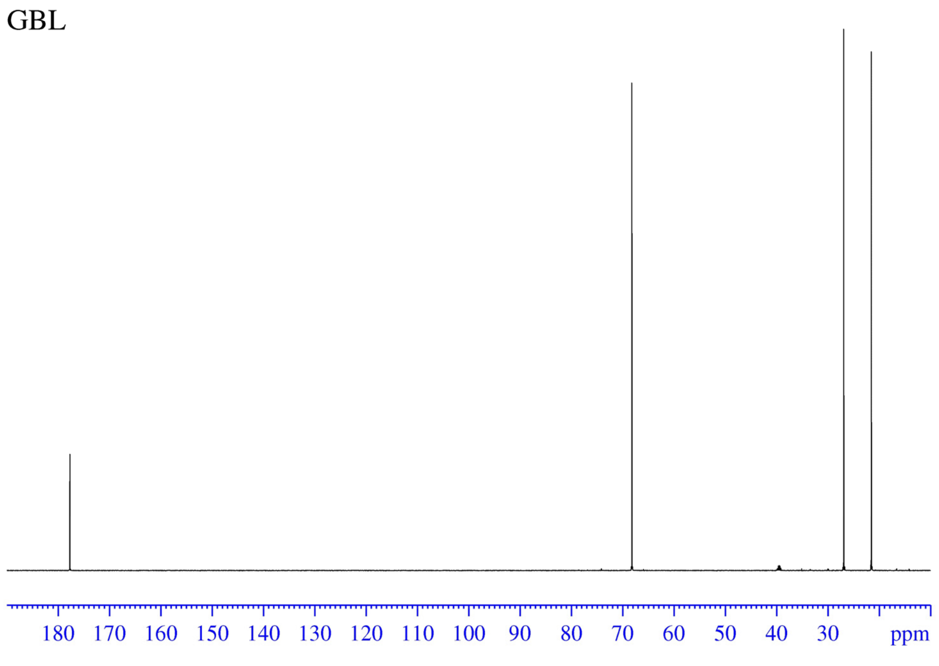

b

FigureS1. ^{1}H NMR (a) and ^{13}C NMR (b) high resolution spectrumof GBL

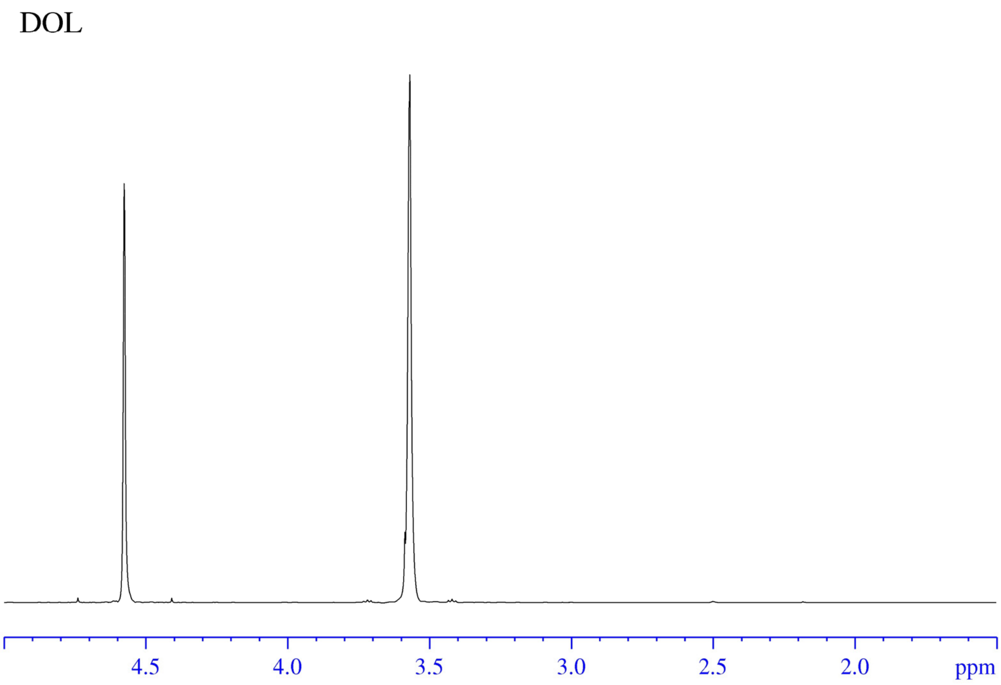

a

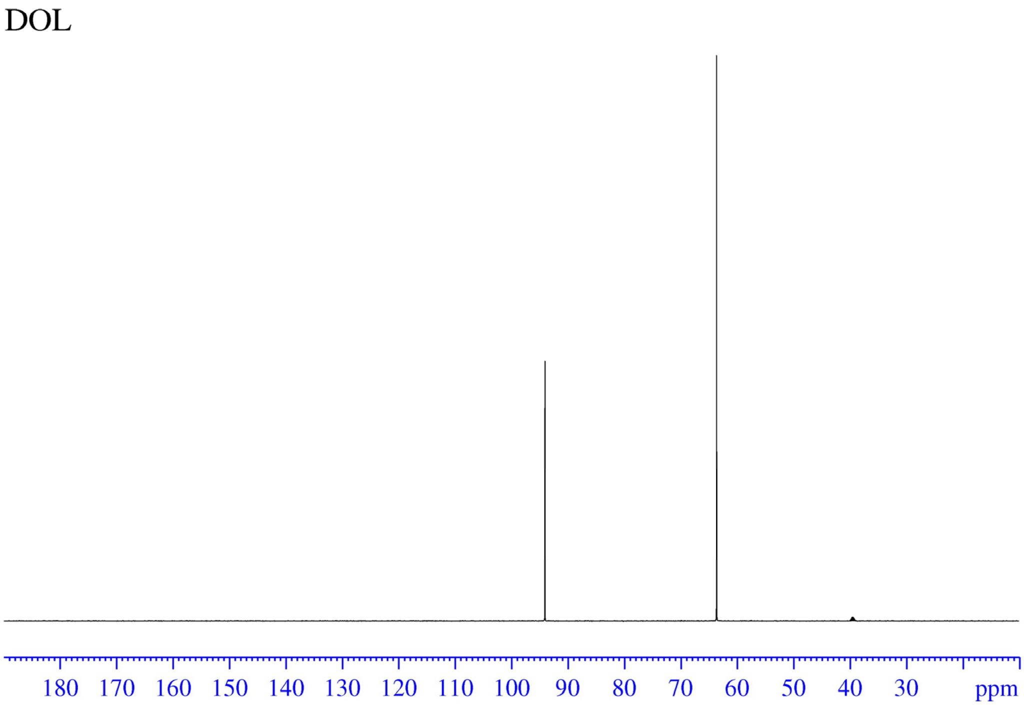

b

FigureS2. ^{1}H NMR (a) and ^{13}C NMR (b) high resolution spectrumof DOL

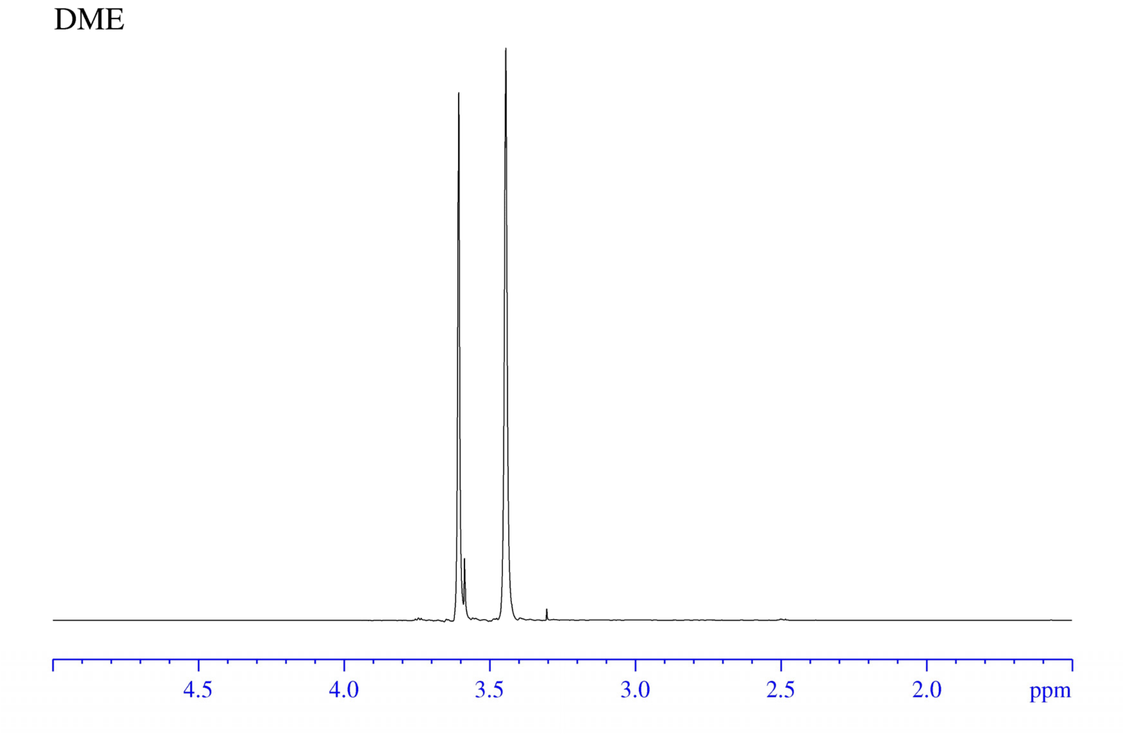

a

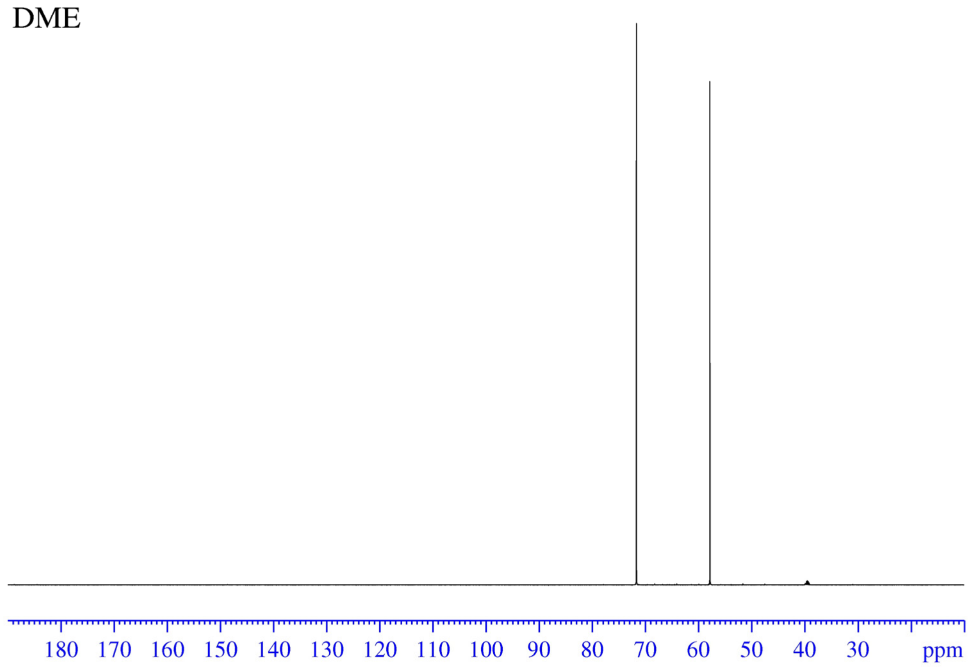

b

FigureS3. ^{1}H NMR (a) and ^{13}C NMR (b) high resolution spectrum of DME

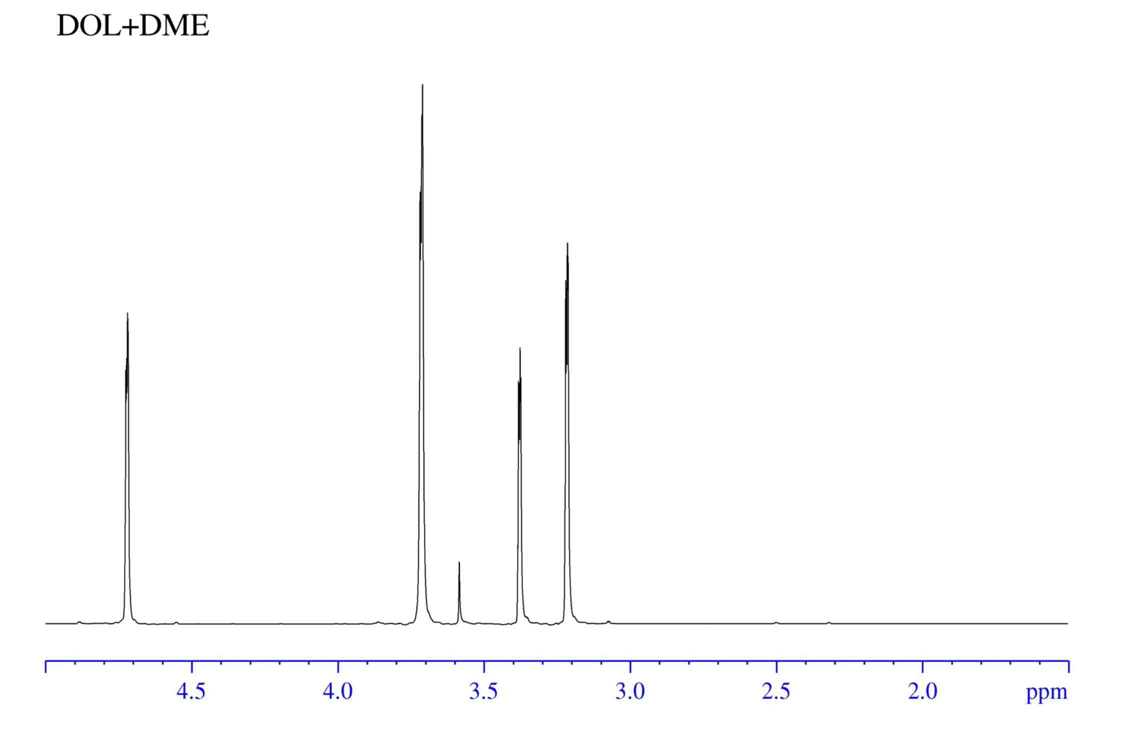

a

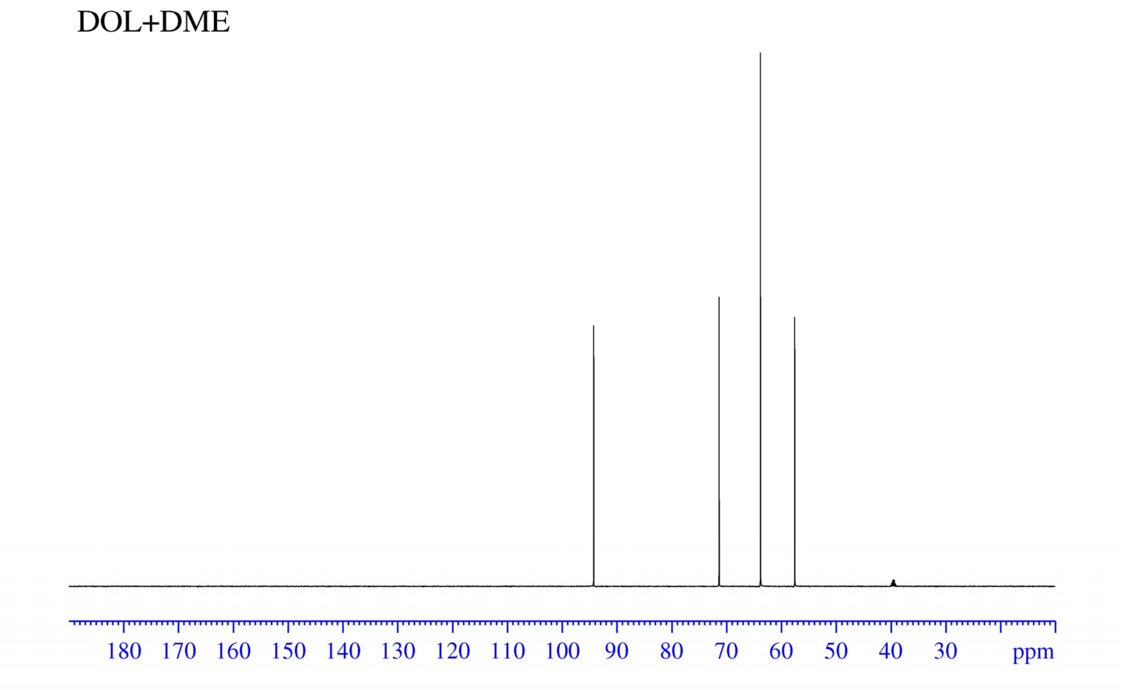

b

FigureS4. ^{1}H NMR (a) and ^{13}C NMR (b) high resolution spectrum of DOL+DME

Assignment of NMR lines of solvent spectra

GBL - 1H (δ ppm, J Hz): 4.09 (2H, t., -CH2-O-, $^3J_{H-H}$=7,0), 2.23 (2H, t., -CH2-CO, $^3J_{H-H}$=8,1), 2.02 (2H, quintet., CH2-C<u>H</u>2-CH2, $^3J_{H-H}$=7,6)

13C (δ ppm, J Hz):177.7 (1C, s., C=O), 68.2 (1C, s., CH2-O), 26.9 (1C, s., <u>C</u>H2-CO), 21.5 (1C, s., CH2-<u>C</u>H2-CH2).

DOL - 1H (δ ppm, J Hz): 4.57 (2H, s., -O-CH2-O-), 3.44 (4H, s., O-CH2-CH2-O);

13C (δ ppm, J Hz): 94.1 (1C,. s, -O-CH2-O-), 63.8 (2C, s., O-CH2-CH2-O);

DME - 1H (δ ppm, J Hz): 3.61 (4H, s., -CH2-O-), 3.44 (6H, s., -CH3-O);

13C (δ ppm, J Hz): 71.7 (2C, s., -CH2-O-), 57.8 (2C, s., O-CH3)

DOL/DME (1:1 mol) - 1H (δ ppm, J Hz): **DOL -** 4.72 (2H, s., -O-CH2-O-), 3.71 (4H, s., O-CH2-CH2-O); **DME-** 3.38 (4H, s., -CH2-O-), 3.22 (6H, s., -CH3-O)

13C (δ ppm, J Hz): **DOL -** 94.2 (1C, s., -O-CH2-O-), 63.8 (2C, s., O-CH2-CH2-O); **DME -** 71.4 (2C, s., -CH2-O-), 57.6 (2C, s., O-CH3)

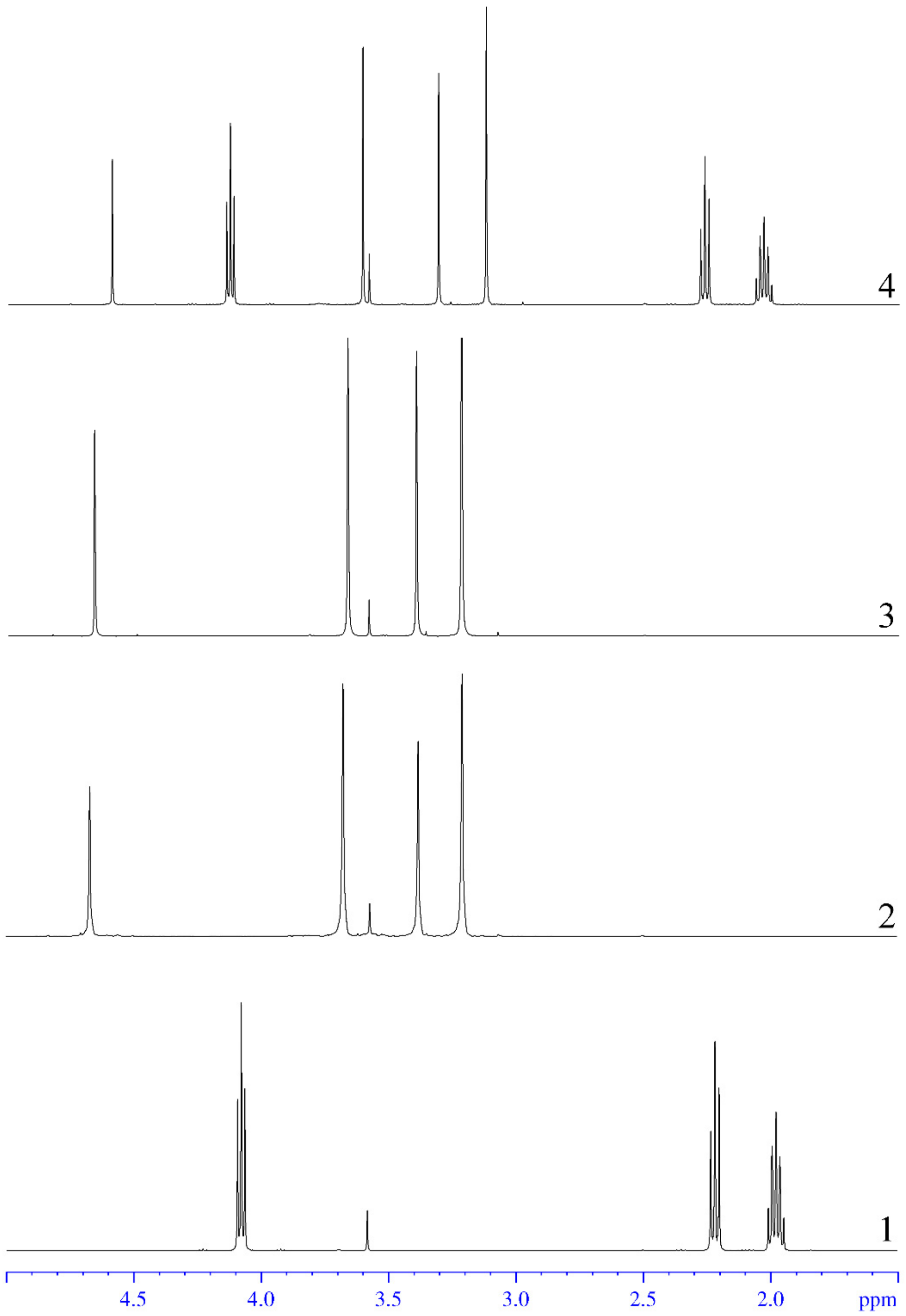

Figure S5. ^{1}H NMR spectrum of electrolytes.The numbers in the figure correspond to the electrolyte composition number.

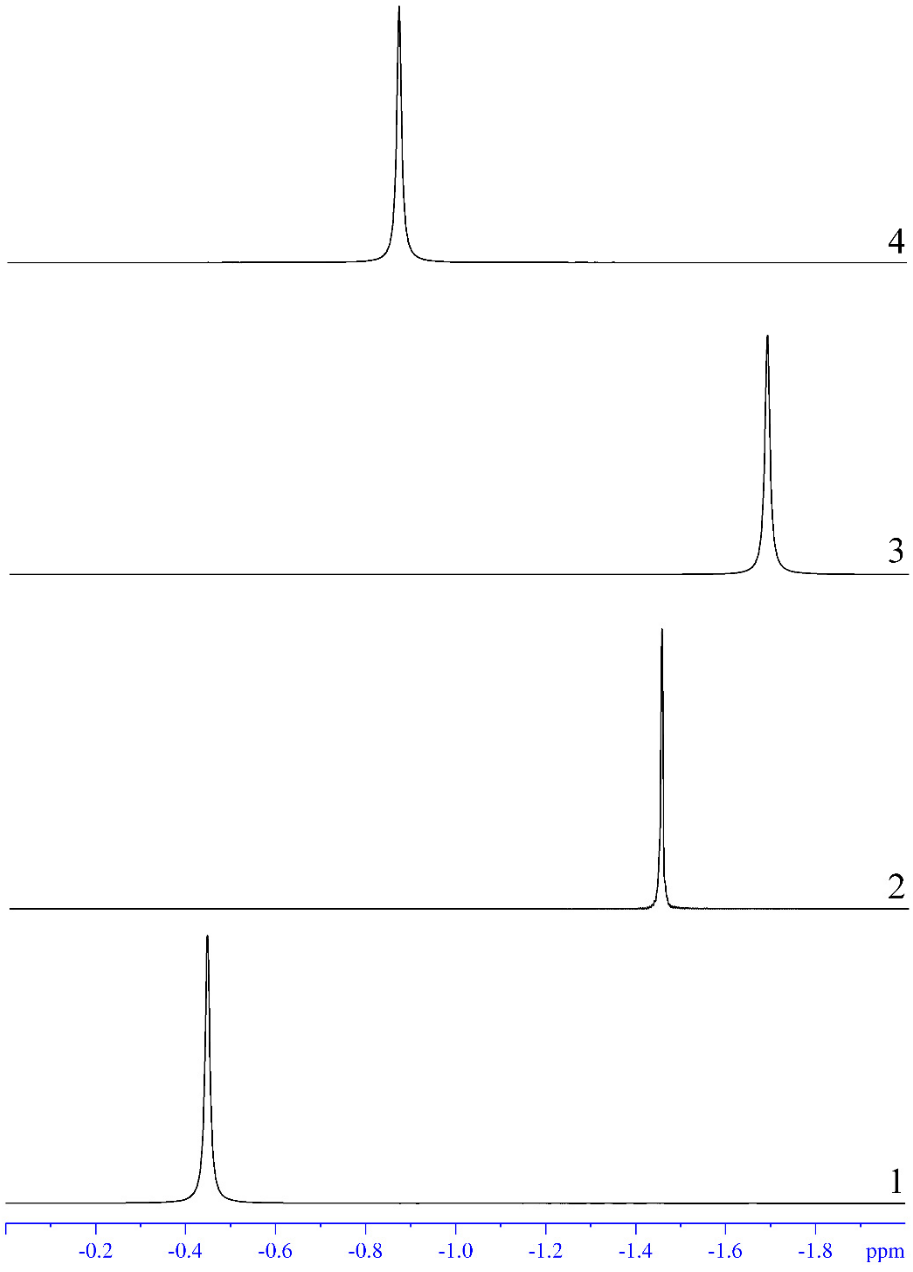

FigureS6. ^{7}Li NMR spectrum of electrolytes. The numbers in the figure correspond to the electrolyte composition number.

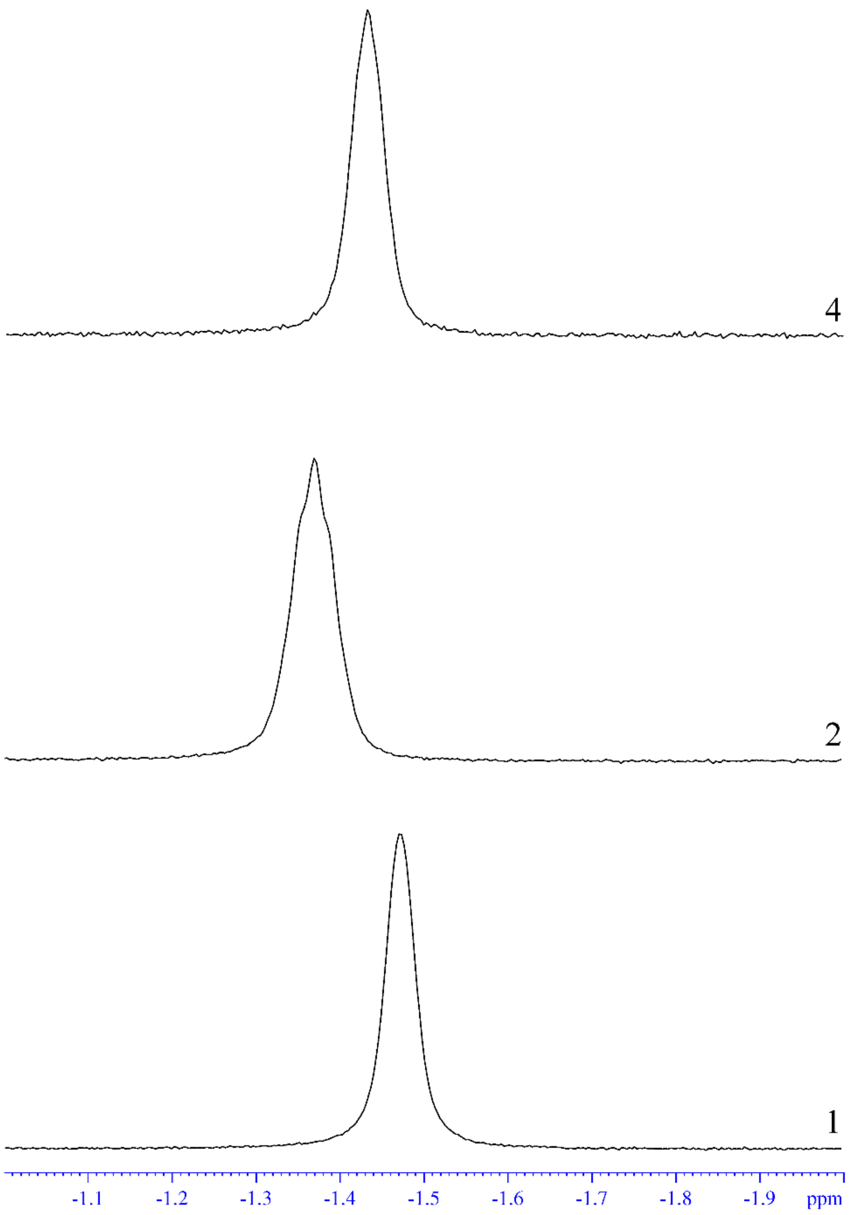

Figure S7. ^{11}B NMR spectrum of electrolytes. The numbers in the figure correspond to the electrolyte composition number.

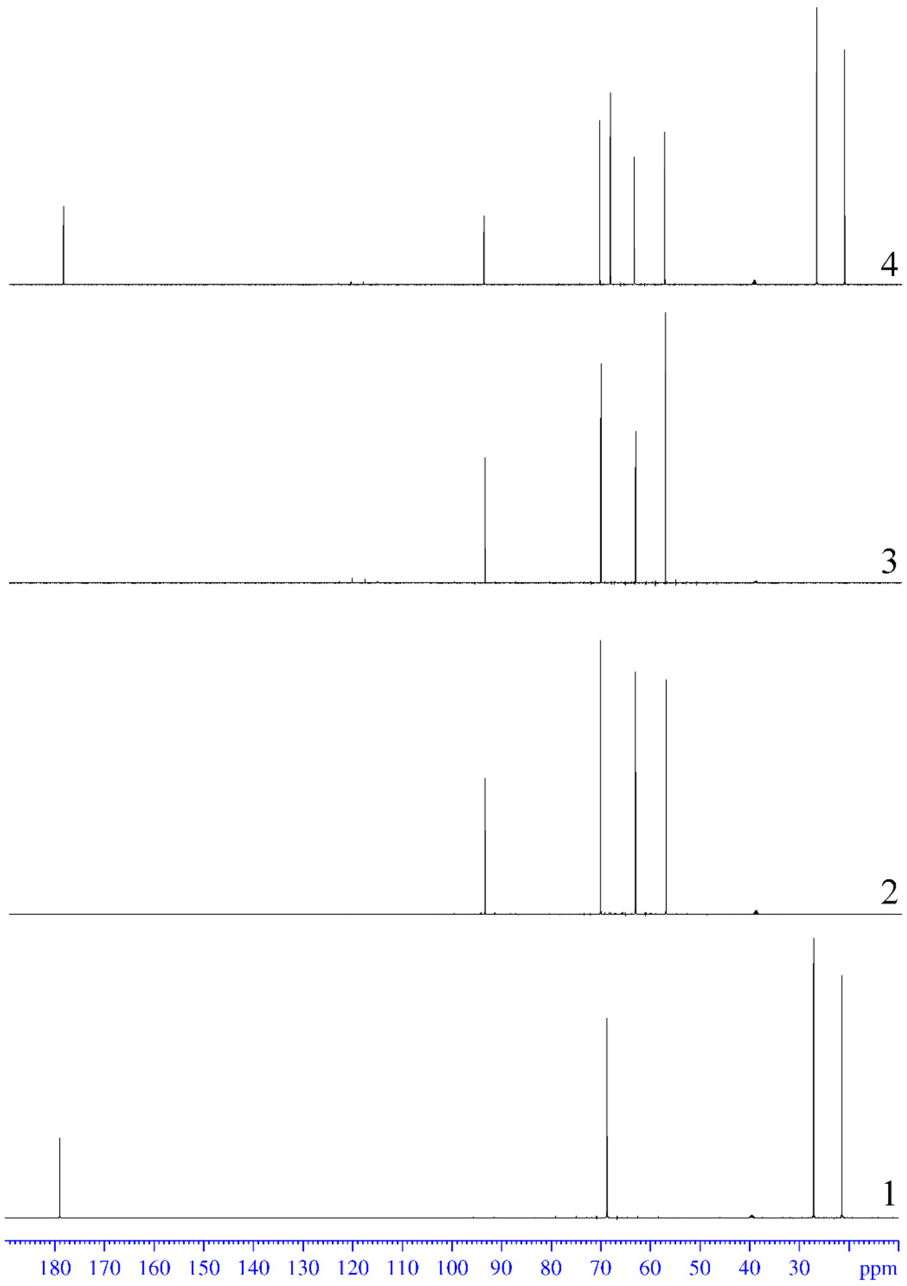

Figure S8. ^{13}C NMR spectrum of electrolytes. The numbers in the figure correspond to the electrolyte composition number.

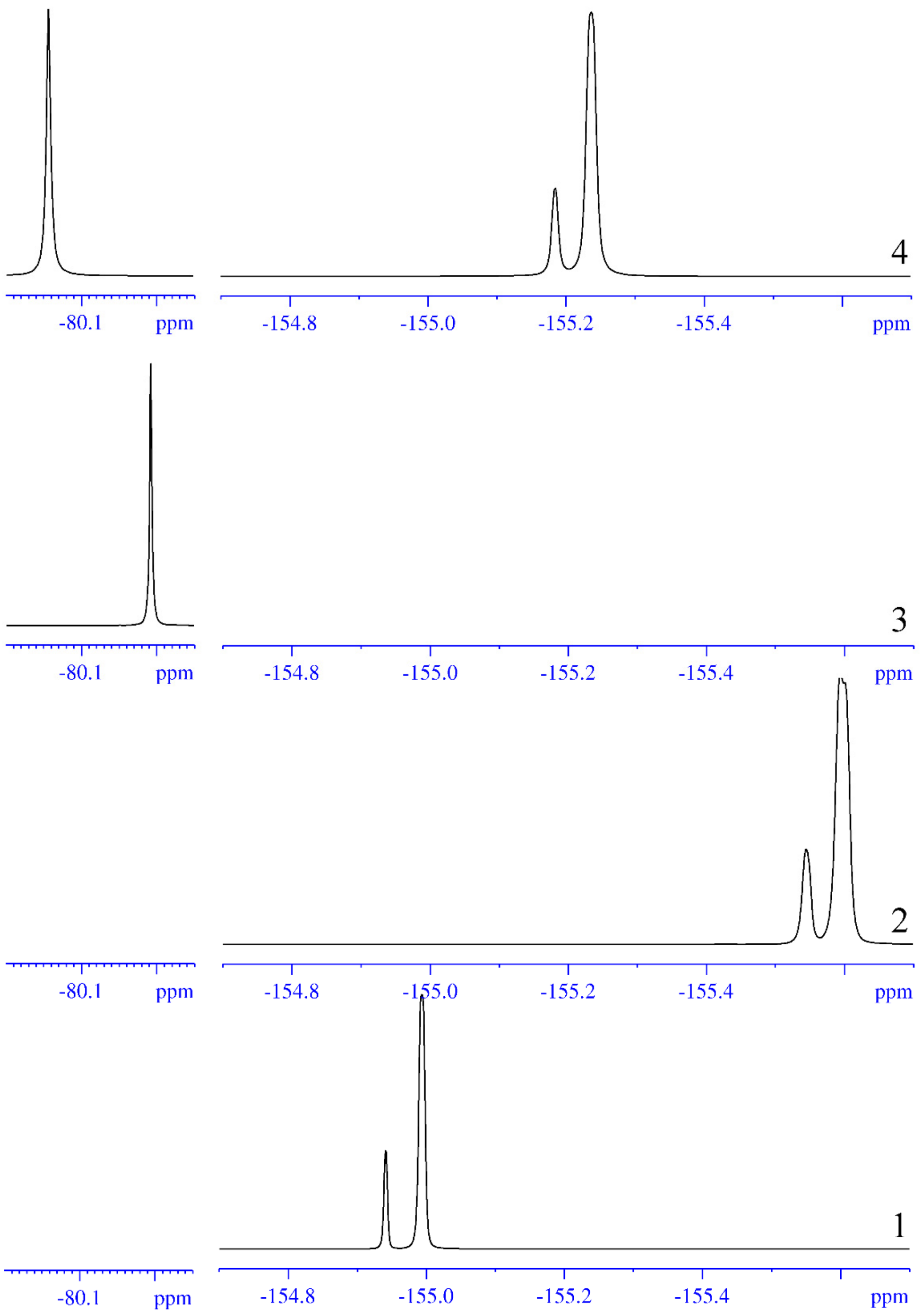

Figure S9. ^{19}F NMR spectrum of electrolytes. The numbers in the figure correspond to the electrolyte composition number.

Assignment of NMR lines of electrolyte spectra

Electrolyte №1 (1 M $LiBF_4$ in GBL)

1H (δ ppm, J Hz): **GBL -** 4.08 (2H, t., -CH2-O-, $^3J_{H-H}$=7,0), 2.22 (2H, t., -CH2-CO, $^3J_{H-H}$=8,1), 1.98 (2H, quintet., CH2-C<u>H</u>2-CH2, $^3J_{H-H}$=7,6)

13C (δ ppm, J Hz): **GBL -** 179.0 (1C, s., C=O), 68.7 (1C, s., CH2-O), 27.1 (1C, s., <u>C</u>H2-CO), 21.4 (1C, s., CH2-<u>C</u>H2-CH2).

7Li (δ ppm): **<u>Li</u>BF4** - -0.45

11B (δ ppm): **Li<u>B</u>F4** -1.47

19F (δ ppm): **LiB<u>F</u>4** - -154.94 (s., $Li^{10}BF4$), -154.99 (s., $Li^{11}BF4$)

Electrolyte №2 (1 M $LiBF_4$ in DOL/DME (1:1v/v))

1H (δ ppm, J Hz): **DOL -** 4.67 (2H, s., -O-CH2-O-), 3.68 (4H, s., O-CH2-CH2-O); **DME-** 3.38 (4H, s., -CH2-O-), 3.21 (6H, s., -CH3-O)

13C (δ ppm, J Hz): **DOL -** 94.1 (1C, s., -O-CH2-O-), 63.8 (2C, s., O-CH2-CH2-O); **DME -** 70.8 (2C, s., -CH2-O-), 57.6 (2C, s., O-CH3)

7Li (δ ppm): **<u>Li</u>BF4** - -1.45

11B (δ ppm): **Li<u>B</u>F4** -1.37

19F (δ ppm): **LiB<u>F</u>4** - -154.94 (s., $Li^{10}BF4$), -154.99 (s., $Li^{11}BF4$)

Electrolyte №3 (1 M LiTFSI in DOL/DME (1:1 v/v))

1H (δ ppm, J Hz): **DOL -** 4.66 (2H, s., -O-CH2-O-), 3.67 (4H, s., O-CH2-CH2-O); **DME-** 3.40 (4H, s., -CH2-O-), 3.22 (6H, s., -CH3-O)

13C (δ ppm, J Hz): **DOL -** 94.1 (1C, s., -O-CH2-O-), 63.8 (2C, s., O-CH2-CH2-O); **DME -** 70.7 (2C, s., -CH2-O-), 57.7 (2C, s., O-CH3);**LiTFSI -** 119.6 (2C, quartet, CF3, $^1J_{C-F}$=321.2)

7Li (δppm): **<u>Li</u>TFSI** - -1.69

19F (δppm): **LiT<u>F</u>SI** - -80.19 (s., CF_3)

<u>Electrolyte №4 (1 M $LiBF_4$ in GBL + 1M LiTFSI in DOL/DME (1:1 v/v))</u>

1H (δppm, J Hz): **GBL -** 4.13 (2H, t., -CH2-O-, $^3J_{H-H}$=7,0), 2.27 (2H, t., -CH2-CO, $^3J_{H-H}$=8,1), 2.03 (2H, quintet., CH2-C<u>H</u>2-CH2, $^3J_{H-H}$=7,6); **DOL -** 4.59 (2H, s., -O-CH2-O-), 3.61 (4H, s., O-CH2-CH2-O);**DME-** 3.31 (4H, s., -CH2-O-), 3.12 (6H, s., -CH3-O)

13C (δppm, J Hz): **GBL -** 179.0 (1C, s., C=O), 68.8 (1C, s., CH2-O), 27.3 (1C, s., <u>C</u>H2-CO), 21.6 (1C, s., CH2-<u>C</u>H2-CH2); **DOL -** 94.3 (1C, s., -O-CH2-O-), 64.0 (2C, s., O-CH2-CH2-O); **DME -** 71.0 (2C, s., -CH2-O-), 57.9 (2C, s., O-CH3)

7Li (δppm): **<u>Li</u>BF4**and**<u>Li</u>TFSI** - -0.87

11B (δ ppm): **Li<u>B</u>F4** -1.43

19F (δ ppm): **LiB<u>F</u>4** -155.18 (s., $Li^{10}BF_4$), -155.24 (s., $Li^{11}BF_4$); **LiT<u>F</u>SI** - -80.06 (s., CF_3)

Table S3. Calculated and corrected values of ^{1}H chemical shifts of solvent molecules for different model solvate complexes in ppm

GBL solvent				DOL/DME solvent							
$LiBF_4$		$(Li^+)(BF_4^-)$		$LiBF_4$		$(Li^+)(BF_4^-)$		LiTFSI		$(Li^+)(TFSI^-)$	
calc.	corr.	calc.	corr.	calc.	corr.	calc.	corr.	calc.	corr.	calc.	corr.
2.13	1.78	2.18	1,82	3,384	3,46	3,493	3,55	3,420	3,49	3,463	3,53
2.90	2.45	2,79	2.35	3.597	3.55	3.571	3.61	3.591	3.63	3.549	3.59
4.53	3.86	4.52	3.85	3.839	3.63	4.070	3.72	4.086	3.73	4.150	3.78
				5.303	4.65	5.458	4.76	5.214	4.58	5.322	4.66
	(0.21)*		(0.17)*		(0.16)*		(0.24)*		(0.18)*		(0.19)*

*The mean square root deviation is given in parenthesis

Table S4. Energies of formation at T=0 K, dE, and enthalpiesof formation dH at T=298 K of solvate complexes

Complex	dE, kcal/mol	dH, kcal/mol
$(Li^+)(DME)_2$	-83.38	-84.34
(Li^+) $(DME)_3$	-99.65	-100.18
(Li^+) $(DME)_{3}z$	-103.21	-104.02
(Li^+) $(DME)_4$	-105.26	-105.65
(Li^+) $(DOL)(DME)_2$	-94.42	-95.11
(Li^+) $(DOL)_2(DME)$	-89.12	-89.71
(Li^+) $(DOL)_2(DME)_2$	-98.65	-98.63
(Li^+) $(DOL)_3$	-76.69	-77.13
(Li^+) $(DOL)_3(DME)$	-96.15	-96.23
(Li^+) $(DOL)_4$	-93.39	-93.64
(Li^+) $(DOL)_5$	-98.00	-97.57
(Li^+) $(GBL)_2$	-77.15	-78.69
(Li^+) $(GBL)_3$	-99.58	-99.50
(Li^+) $(GBL)_4$	-114.04	-113.35

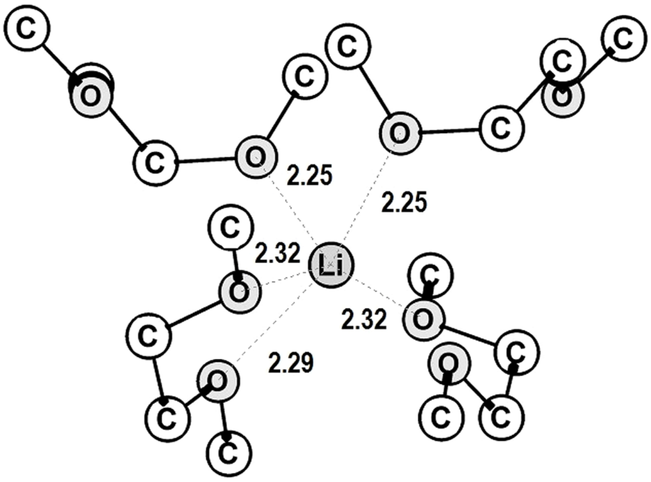

2.25
2.25
2.32
Li
2.32
2.29

a

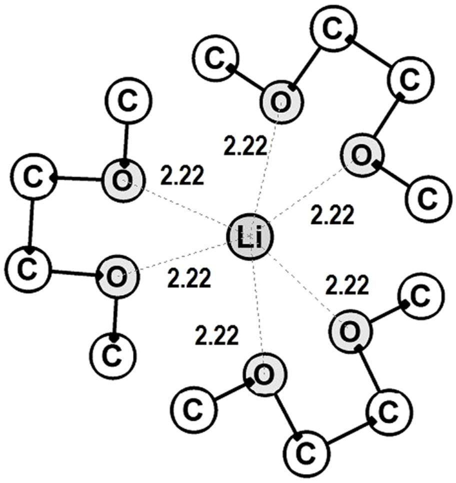

2.22
2.22
Li
2.22
2.22
2.22
2.22

b

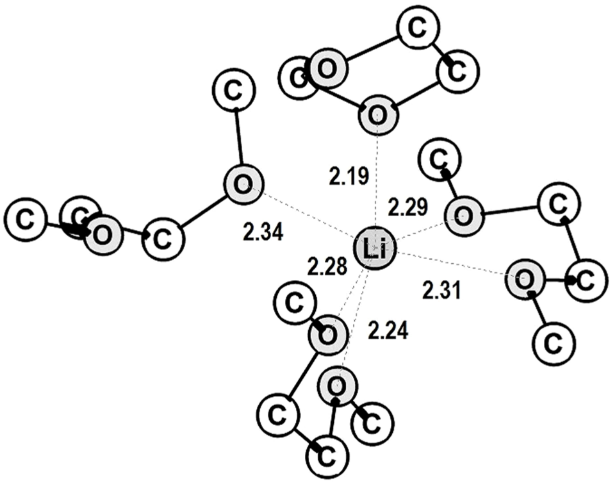

2.19
2.29
2.34
Li
2.28
2.31
2.24

c

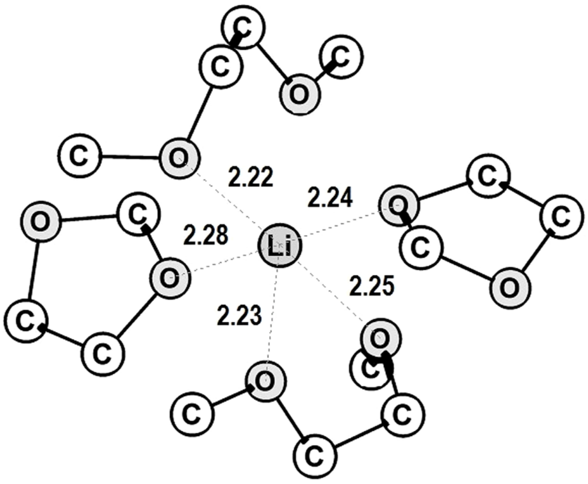

2.22
2.24
2.28
Li
2.25
2.23

d

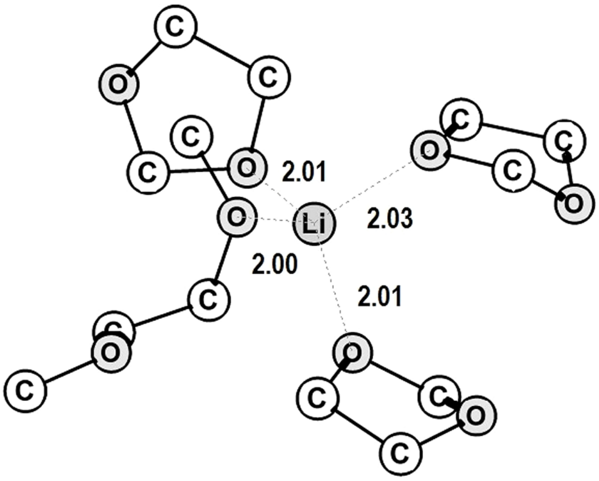

2.01
Li
2.03
2.00
2.01

e

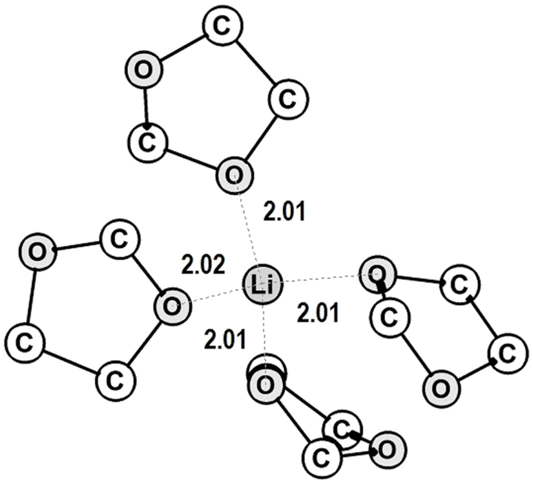

2.01
2.02
Li
2.01
2.01

f

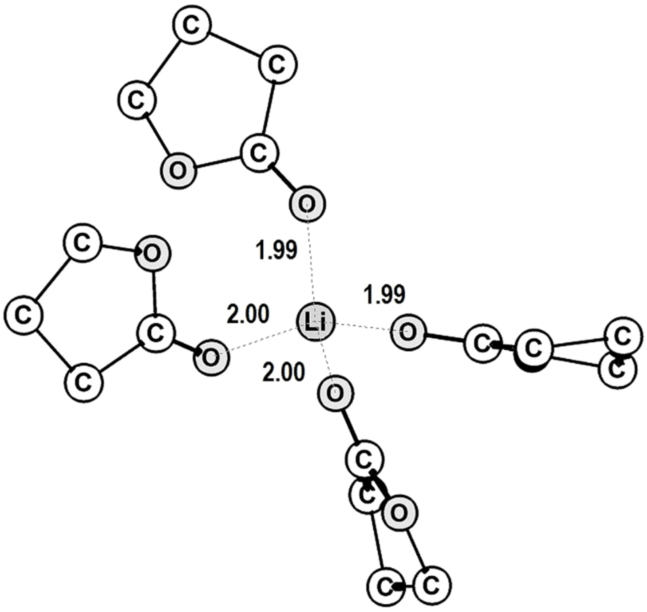

g

Figure S10. Calculated structure of solvate complex $Li^{+}(DME)_4$ (a), $Li^{+}(DME)_3$ (b), $Li^{+}(DOL)(DME)_3$ (c), $Li^{+}(DOL)_2(DME)_2$ (d), $Li^{+}(DOL)_3(DME)$ (e), $Li^{+}(DOL)_4$ (f), $Li^{+}(GBL)_4$ (g). Bond lengths are given in Å

Table S5. Cartesian coordination of calculated structures in Å

$LiBF_4(GBL)_{12}$

6	3.25057418	-0.14383626	-3.21068241
6	4.81370153	-0.70632594	-1.67888884
6	4.53552017	-0.29470200	-3.98272362
8	5.25979923	-1.05184684	-0.62399794
1	3.00254352	0.89663907	-3.03223414
1	2.39473523	-0.63390315	-3.65596091
1	4.65264199	0.46123496	-4.76285385
1	4.56952281	-1.27905324	-4.44766607
6	-4.66122422	-1.15913945	4.25213988
6	-2.44447954	-0.42003763	4.06141969
6	-4.45799053	-0.30467951	3.02908804
8	-3.17133019	0.26105236	3.19517662
8	-1.33859273	-0.10995171	4.39849840
1	-5.04526838	-0.55074810	5.07385743
1	-5.35639901	-1.98309357	4.08098460
1	-5.17298492	0.50966560	2.92940567
1	-4.45426405	-0.89962429	2.12116948
6	3.66353994	4.23578680	1.11208947
6	4.20512177	2.99652536	-0.71046980
6	4.86836207	4.90818109	0.49722210
8	4.22755054	2.05697144	-1.45066990
1	3.76009562	4.04726919	2.18212189
1	2.74168432	4.77714419	0.93404098
1	5.77669278	4.60580778	1.02381151
1	4.80804562	5.99845811	0.53269109
8	3.57367223	2.98949213	0.45073038
8	3.51156926	-0.74851859	-1.96163603

8	0.84300753	4.62012265	0.09587390
6	0.87389600	4.23930268	-1.03479525
8	2.01531689	3.94227368	-1.66602228
6	0.33416301	3.17550580	-3.06082145
6	1.77988405	3.59793353	-3.01706545
1	0.24977594	2.13302914	-2.77353215
1	-0.12249479	3.30691122	-4.04351338
1	1.96811105	4.48223494	-3.63547070
1	2.49283792	2.82624277	-3.30144961
6	3.10349050	0.72627488	2.28128006
6	1.59560400	1.33011027	0.58160939
6	1.95986637	1.56622578	2.78911310
8	1.33210019	2.07354868	1.62658941
8	1.04599838	1.47334982	-0.48213186
1	4.00127555	1.33029960	2.18248629
1	3.33175092	-0.10714502	2.94275277
1	2.26560778	2.41355706	3.40678766
1	1.22391561	0.98300572	3.33696537
6	-0.26132028	4.05393253	-1.99567915
1	-0.51710445	5.04430975	-2.38200499
1	-1.13473072	3.65616849	-1.49985565
6	4.86185769	4.33503395	-0.89798772
1	4.22551753	4.90398302	-1.57321986
1	5.84198301	4.22054105	-1.36163423
6	5.55772203	-0.19834669	-2.87999418
1	6.46856393	-0.77811599	-3.03284722
1	5.83294499	0.83225191	-2.66264553
6	-3.24941317	-1.59404805	4.53706112
1	-2.97138719	-2.43668862	3.91424358

1	-3.02313440	-1.82563534	5.57716284
6	2.60773107	0.29488002	0.93118353
1	2.09099794	-0.65350808	0.96973162
1	3.35701130	0.24675825	0.15567158
9	-1.66317847	-1.56574909	1.95484297
5	-0.66842410	-0.71974218	1.52993280
9	-1.05692078	0.58930504	1.54102554
9	-0.33200550	-1.05309686	0.19860230
3	-0.24820036	0.20764065	-1.10203149
9	0.45791672	-0.89788295	2.30186695
8	-3.17458627	2.87430082	-3.80074112
6	-3.27703176	1.69380070	-3.67812958
8	-4.14968207	1.09631829	-2.85514522
6	-4.04429755	-0.31651599	-2.92122671
1	-5.04691262	-0.74117988	-2.83711852
1	-3.45719505	-0.66954247	-2.07593323
6	-2.47857711	0.62388138	-4.37163642
1	-2.23888928	0.92227359	-5.39173688
1	-4.08741458	-0.62492796	-5.04672648
1	-1.55113587	0.52124564	-3.81104597
6	-3.35574076	-0.59439467	-4.23605361
1	-2.81760794	-1.54256110	-4.23451269
8	3.39934538	-3.21477860	-3.56753615
6	3.07103741	-3.35121580	-2.42186890
8	1.85777828	-3.03968731	-1.98304099
6	1.71592964	-3.32436367	-0.59579207
1	1.20291022	-4.28057901	-0.50447446
1	1.09456128	-2.56043029	-0.15422031
6	3.87224906	-3.89865550	-1.27796747

1	3.83444577	-4.98918702	-1.35958274
1	3.20767310	-4.01890558	0.80946154
1	4.91247208	-3.59231560	-1.34854336
6	3.12314377	-3.38881977	-0.07399019
1	3.47700450	-2.39619418	0.17852954
8	-1.76590441	1.26470169	-1.21555056
6	-2.74332887	1.76327860	-0.71162778
8	-3.06988516	3.00980490	-0.93485665
6	-4.36128706	3.32830639	-0.43341374
1	-4.93217399	3.75514991	-1.26124628
1	-4.24349314	4.08328290	0.33932888
6	-3.73388566	1.13175370	0.19255381
1	-3.90150067	0.10028348	-0.08457951
1	-5.65446297	1.62422768	-0.62283262
1	-3.28307872	1.13655127	1.17354393
6	-4.93622657	2.03108161	0.08984275
1	-5.44242917	2.17334163	1.04439015
8	-3.97270609	-1.92505670	0.12148512
6	-3.39575174	-2.85011829	0.61623865
8	-3.66165853	-3.31621203	1.83670513
6	-2.81253661	-4.40860651	2.15292062
1	-3.38124507	-5.11773982	2.76096228
1	-1.97171904	-4.03827793	2.72962256
6	-2.35237689	-4.94513226	0.82372928
1	-1.39711081	-5.46404391	0.89195898
1	-3.09767389	-5.63575246	0.42023207
1	-2.47776056	-3.79997307	-1.05836039
6	-2.30902120	-3.68164162	0.00735277
1	-1.38496985	-3.13705720	0.15625833

8	-4.27305804	2.77145516	3.48514172
6	-3.13868759	2.98107048	3.16579111
8	-2.77309533	3.23040592	1.90994368
6	-1.39523674	3.54818200	1.81067362
1	-1.28305851	4.36791870	1.10843733
1	-0.87430063	2.68455332	1.43061700
6	-0.96933827	3.87802665	3.21517981
1	0.08631637	3.67263011	3.38366747
1	-1.14446300	4.93515001	3.42654687
1	-2.14034538	3.38129545	5.02406504
6	-1.90867484	3.02005540	4.02295293
1	-1.55345943	1.99485788	4.09091702
8	0.26073498	-3.77424618	1.90115566
6	0.79570825	-3.29314748	2.85668316
8	2.09064525	-2.97603359	2.89127023
6	2.46919172	-2.54606640	4.18857626
1	3.23971974	-1.78035265	4.09078599
1	2.91190854	-3.39768474	4.71667044
6	1.19014376	-2.08317259	4.84008695
1	1.22240884	-2.15991732	5.92949293
1	0.97176900	-1.05513569	4.57489452
1	-0.79372858	-2.55449399	4.05760372
6	0.18365797	-2.99167036	4.19175326
1	0.07785316	-3.94188870	4.72322530
8	0.16191480	-0.65932038	-2.74410658
6	-0.02072902	-1.75993478	-3.19640454
8	-0.76906472	-2.66869106	-2.60232046
6	-0.73932841	-3.89901015	-3.31618827
1	-1.72142433	-4.36971882	-3.22732090

1	-0.00049166	-4.54758650	-2.84732845
6	-0.35361581	-3.52245121	-4.72738521
1	0.16365009	-4.32880593	-5.25193346
1	-1.24674501	-3.26693536	-5.30214960
1	0.44022881	-1.53718254	-5.27333972
6	0.49912935	-2.29887138	-4.49619007
1	1.54319876	-2.55654306	-4.33583493

$Li^{+}BF_4^{-}(GBL)_{12}$

6	3.44336903	0.36704319	-2.57807239
6	4.71050084	-0.29632637	-0.82726131
6	4.87094569	0.43882143	-3.05283453
8	4.97062114	-0.79026613	0.23155153
1	3.06392618	1.35102573	-2.33226872
1	2.74939112	-0.11762421	-3.25120987
1	5.05781387	1.29493337	-3.70469410
1	5.13074722	-0.47369795	-3.58801778
6	-3.40407062	-0.27544271	4.77560350
6	-1.22587723	0.48331605	4.29644076
6	-3.19809326	-0.01292416	3.30773038
8	-1.98334808	0.71181301	3.22799426
8	-0.12484514	0.93077194	4.43323378
1	-3.90494158	0.57532663	5.24455844
1	-4.00054773	-1.16848975	4.95821888
1	-3.98533501	0.58935925	2.85370219
1	-3.08802222	-0.92952188	2.75103423
6	2.95785550	5.42265567	1.15308188
6	3.49826476	4.17277872	-0.67277369

6	3.60139014	6.37308281	0.16771992
8	3.76893877	3.20853263	-1.32893798
1	3.39762145	5.45392884	2.15328033
1	1.88499921	5.57844721	1.23163424
1	4.65628467	6.51476349	0.41447342
1	3.12355051	7.35533561	0.15607679
8	3.18268095	4.12709881	0.61344719
8	3.50961716	-0.38896692	-1.38900013
8	0.23084533	5.05997212	-0.06410163
6	0.26109627	4.52478859	-1.13410093
8	0.89755856	3.38284316	-1.35470373
6	-0.23910022	3.82437737	-3.32161481
6	0.91456997	3.05897780	-2.73012150
1	-1.14130815	3.23827715	-3.22520454
1	-0.08993753	4.06465699	-4.37669194
1	1.87567416	3.37825204	-3.13731155
1	0.83718084	1.98243109	-2.83609772
6	1.79594761	1.76750732	2.06309349
6	0.93539605	0.98228985	0.03130503
6	0.48162733	2.40243787	1.72457858
8	-0.01762411	1.64717094	0.63589903
8	0.74384146	0.35388183	-0.97744935
1	2.50730582	2.47798674	2.48076116
1	1.62964220	0.97869763	2.78170776
1	0.59070132	3.42547014	1.38192929
1	-0.24334229	2.35407732	2.52885563
6	-0.31419146	5.02758806	-2.42417275
1	0.32518172	5.84530696	-2.76946281
1	-1.31383369	5.42031913	-2.26515158

6	3.47010005	5.60811250	-1.12597659
1	2.50610508	5.77931127	-1.60071245
1	4.24903168	5.79227865	-1.86552204
6	5.60165109	0.50318904	-1.73563728
1	6.61508577	0.10217103	-1.72896853
1	5.62264189	1.51995858	-1.34019549
6	-1.98517728	-0.37952747	5.26138473
1	-1.60358493	-1.38634326	5.15583875
1	-1.81288047	-0.04165718	6.28334875
6	2.22221228	1.19342008	0.74396180
1	2.77035209	0.26577620	0.79774357
1	2.78866388	1.90078171	0.15157225
9	-0.69479897	-1.80784555	3.18454418
5	-0.41299688	-1.69048141	1.83076974
9	-1.47519232	-1.09346697	1.17638074
9	-0.21867622	-2.95790302	1.31706795
3	-0.62743418	-0.10869724	-2.17653483
9	0.71743910	-0.91840831	1.67432843
8	-3.48450838	4.41957165	-2.64906236
6	-3.87841910	3.31885905	-2.88299340
8	-4.48984026	2.54105405	-1.98310657
6	-4.86767485	1.29301611	-2.54367769
1	-5.84178357	1.01507469	-2.13370143
1	-4.13944818	0.54144365	-2.24546888
6	-3.77069457	2.54897719	-4.17138069
1	-3.84713498	3.21503904	-5.03025833
1	-5.81988017	1.93588583	-4.35893889
1	-2.78343848	2.08506928	-4.17387977
6	-4.86298229	1.51835411	-4.03720801

1	-4.68678367	0.60085406	-4.60335751
8	4.01013538	-2.63584016	-3.24721867
6	3.28920602	-2.82198591	-2.30372023
8	2.01509001	-2.46121221	-2.30145216
6	1.38614541	-2.77155764	-1.06221262
1	0.79302287	-3.67137187	-1.21574147
1	0.72917110	-1.96030725	-0.80665525
6	3.61085692	-3.48287598	-0.99856739
1	3.55309760	-4.56261646	-1.16712631
1	2.24552206	-3.66104578	0.70549455
1	4.62285264	-3.24629296	-0.68123454
6	2.51317718	-2.98420540	-0.09915401
1	2.80082287	-2.03960284	0.33644378
8	-1.74640849	1.37981729	-1.86659695
6	-2.09060807	1.96419717	-0.86877470
8	-1.85839867	3.24210184	-0.71456147
6	-2.44127802	3.75252229	0.46770696
1	-2.89370832	4.71476758	0.22838628
1	-1.64441846	3.91156140	1.18885856
6	-2.84682726	1.44656099	0.30132088
1	-3.56927644	0.70846974	-0.01679107
1	-4.40412113	2.90817012	0.49146349
1	-2.13225429	0.95246999	0.94585341
6	-3.42138710	2.69802090	0.91018882
1	-3.50994998	2.65373243	1.99536178
8	-3.26187337	-2.97991519	2.46876890
6	-2.66035027	-3.66183133	3.24651858
8	-2.57628430	-3.39013674	4.54463633
6	-1.85891219	-4.40314593	5.23820649

1	-2.35433381	-4.56914100	6.19947672
1	-0.84899770	-4.04757118	5.41708578
6	-1.86885167	-5.59757132	4.31723698
1	-0.99833460	-6.23787656	4.45688768
1	-2.76915009	-6.19432268	4.48810719
1	-2.43478696	-5.50525532	2.18884492
6	-1.92715803	-4.93538443	2.96536479
1	-0.94240024	-4.64869969	2.61176333
8	-1.41104727	-1.67860795	-1.59749575
6	-2.37884565	-2.10310967	-1.03126679
8	-3.47654435	-1.38775732	-0.83848910
6	-4.45807467	-2.14804629	-0.13528423
1	-5.44136626	-1.86443181	-0.52118710
1	-4.39561284	-1.89380110	0.91809072
6	-4.07763627	-3.58498261	-0.38010192
1	-4.42304487	-4.24710804	0.41273754
1	-4.50160119	-3.93005528	-1.32706688
1	-2.09193576	-4.21347640	-1.12288517
6	-2.57795481	-3.47806892	-0.48387781
1	-2.09787124	-3.47967774	0.48631691
8	0.79554114	-4.31405174	3.85405856
6	1.45334130	-3.31469219	3.89122564
8	2.24909531	-2.91723491	2.91128132
6	3.01658408	-1.79059223	3.29347781
1	3.17322823	-1.16102814	2.42258290
1	3.99549094	-2.14662295	3.63135252
6	2.23179497	-1.15210022	4.40874208
1	2.85897278	-0.58253953	5.09754637
1	1.48089448	-0.49008509	4.00551984

1	0.58458038	-2.15138646	5.46080495
6	1.56289720	-2.34466536	5.03339891
1	2.18240035	-2.81960695	5.79994230
8	0.35065784	-0.45144897	-3.76613181
6	0.61326106	-1.51935767	-4.26111580
8	-0.08542388	-2.61230354	-4.03487218
6	0.51082255	-3.73729082	-4.66960823
1	-0.28810705	-4.41244237	-4.98734209
1	1.12985405	-4.25326342	-3.93645872
6	1.33148817	-3.15623938	-5.79691547
1	2.18001893	-3.78701654	-6.07082091
1	0.70586066	-3.02543461	-6.68314058
1	1.83518706	-1.00936469	-5.94283048
6	1.72685184	-1.81827212	-5.22092542
1	2.63659052	-1.89341459	-4.63044787

$\underline{LiBF_4(DD)_{12}}$

6	1.26226798	-3.08162189	4.45724064
6	0.44088904	-2.41531181	2.54460962
6	0.49839585	-1.79100259	4.69305162
8	0.21773547	-1.32916825	3.40032519
1	2.18579701	-3.15911331	5.03411053
1	0.64456222	-3.95269678	4.69796782
1	1.08542723	-1.05112348	5.22343859
1	-0.42423298	-1.96039650	5.25627146
8	1.54430147	-3.03129541	3.09018846
6	4.02701446	-0.53124815	4.87954871
6	2.56243377	1.10446778	4.98199634

6	3.37229562	-0.38711484	3.51559678
8	2.32312196	0.51693643	3.74774890
1	4.18608482	-1.56718064	5.18637583
1	4.99485920	-0.02051213	4.91022120
1	2.96157102	-1.31291700	3.14159456
1	4.05715017	0.00443926	2.76552129
8	3.11196507	0.09136966	5.74646925
6	0.19225669	1.85518411	-3.02099174
6	-1.40438756	1.86898204	-1.49870138
6	-0.24765156	0.43708736	-2.80664956
8	-1.20579748	0.53316480	-1.80639441
1	1.08871830	2.06685140	-2.44781354
1	0.39582916	2.11079662	-4.06507071
1	0.56212558	-0.21435739	-2.51220169
1	-0.70670195	0.02931592	-3.70220328
8	-0.88777875	2.59200271	-2.55209421
6	-1.30721211	-2.85744643	-1.81932875
8	-1.05400755	-2.34521143	-3.07994039
6	-2.32232628	-2.11692108	-3.61374485
1	-2.27299094	-1.39850968	-4.43305223
6	-3.09830765	-1.62704429	-2.41944159
8	-2.39365080	-2.14932577	-1.33356445
1	-4.13364901	-1.97705959	-2.41277402
1	-3.10083617	-0.54821432	-2.37053216
1	-2.74740578	-3.04659248	-4.00813912
1	-1.57176796	-3.91951706	-1.88975621
1	-0.44224262	-2.74607421	-1.17363330
1	3.26766337	1.94058763	4.86687397
1	1.63587741	1.48557776	5.42402998

1	-2.45851135	2.09524761	-1.36935194
1	-0.87720796	2.10418987	-0.58174215
1	0.60408982	-2.08771308	1.53093513
1	-0.42728836	-3.07703981	2.58026363
6	5.21074171	-2.29637839	0.21699987
1	4.83826883	-1.27673716	0.20108976
1	6.12253115	-2.35027409	-0.37869602
1	5.46211230	-2.55362061	1.25294204
1	3.07409784	-2.97160022	1.33272086
6	3.00350790	-3.08277045	0.25366127
1	2.48499400	-2.21336407	-0.12815293
8	4.27920598	-3.20219941	-0.32470456
6	2.21713415	-4.32191703	-0.05488900
1	2.31533328	-4.54845495	-1.11979823
8	0.86501426	-4.15324773	0.28713776
1	2.64975760	-5.16910411	0.48525847
6	0.13641290	-5.35974923	0.23430012
1	0.47668688	-6.06998951	0.99569688
1	0.23101149	-5.85100939	-0.73834141
1	-0.91650723	-5.13732013	0.40332658
6	2.40344781	-2.06024525	-2.82741853
1	2.30197397	-1.80201801	-3.88575475
1	1.46129329	-2.47566883	-2.48661867
1	3.18474033	-2.81690471	-2.74209831
1	4.73535912	-0.88625060	-2.19278295
6	3.85909525	-0.27697002	-2.40973474
1	3.87180248	-0.06523129	-3.48834586
8	2.68945362	-0.93135274	-2.04601519
6	3.94718158	1.01253408	-1.66673011

1	4.75797726	1.62229286	-2.07235462
8	4.15800826	0.77339586	-0.31112788
1	3.01580718	1.55212225	-1.81086102
6	4.07169089	1.93052568	0.46965734
1	3.07505568	2.36176572	0.43487594
1	4.78826572	2.69114858	0.15156995
1	4.29044259	1.66305769	1.50292261
6	-1.30392657	1.47226981	4.28277025
1	-1.68286126	2.32117661	4.85856756
1	-0.90194656	0.73764409	4.97848188
1	-2.13054302	1.03189617	3.73268846
1	-1.47209977	2.38172554	1.83060750
6	-0.70645414	2.81605044	2.46311452
1	-1.14827489	3.67092295	2.98154887
8	-0.26805978	1.86380653	3.41147070
6	0.43208991	3.27525829	1.60206471
1	1.10945197	3.91858517	2.17688235
8	-0.10221695	3.94545816	0.50518312
1	1.00411076	2.41122754	1.28850956
6	0.83186194	4.30149750	-0.48402509
1	1.43846754	3.45061832	-0.77884026
1	1.50493458	5.09047187	-0.13002533
1	0.28066775	4.65659169	-1.34663145
3	0.70714253	0.36274506	2.72156650
9	-0.24860814	0.39529606	1.09557855
5	0.85165162	0.02833303	0.30544531
9	0.58603984	-1.13375316	-0.34846396
9	1.21176167	1.06907137	-0.49422417
9	1.83709230	-0.18321291	1.28391429

1	-5.05073995	-5.60335693	-1.39394972
6	-4.05717031	-5.17432054	-1.56561299
1	-4.18322385	-4.27663950	-2.17284103
1	-3.46200895	-5.89789451	-2.12796546
8	-3.38850625	-4.90160678	-0.35876161
6	-4.03944045	-3.94540171	0.43662224
6	-3.20826133	-3.69919809	1.65938578
1	-2.23111297	-3.32466793	1.35114305
1	-5.02808502	-4.30440974	0.74408096
1	-3.03979294	-4.65005083	2.17378917
1	-4.17619918	-3.00856132	-0.09593074
8	-3.85745426	-2.78590484	2.50531240
6	-3.22086164	-2.60976570	3.74608842
1	-2.26672298	-2.08922478	3.64327704
1	-3.03294408	-3.56274193	4.25073260
1	-3.87482484	-2.01087080	4.38364679
1	-3.12652528	1.03882309	-5.74602918
6	-2.76483490	1.73429123	-4.97977449
1	-3.00426830	2.74703870	-5.31603822
1	-1.68681308	1.64367914	-4.90473117
8	-3.31504209	1.45913182	-3.72146411
6	-4.70225368	1.57042138	-3.67112723
6	-5.13329419	1.61591497	-2.23983776
1	-4.76958870	0.73487961	-1.71589777
1	-5.19068085	0.73197166	-4.18993389
1	-6.23121072	1.60138552	-2.18605044
1	-5.03793502	2.48651076	-4.16558179
8	-4.61613336	2.77925633	-1.67215233
6	-4.94644794	2.96692726	-0.32113336

1	-4.47696650	2.21681511	0.30761666
1	-6.02884142	2.91817101	-0.16137337
1	-4.59168349	3.94808443	-0.02160592
8	-4.16371528	-0.47397540	0.06677965
6	-5.12324449	-0.33069371	1.07052440
1	-5.98601372	0.21024828	0.67173521
1	-5.47406792	-1.30014733	1.42866194
6	-4.39434578	0.42591775	2.16599220
1	-4.88053494	1.36181167	2.45608476
1	-4.30683952	-0.19003968	3.06402294
8	-3.13811436	0.69556638	1.61775382
6	-2.92733476	-0.30329701	0.66871277
1	-2.62674699	-1.21842606	1.16884717
1	-2.17885414	-0.01779510	-0.05433578
8	-1.57164069	5.73595499	-2.66519363
6	-1.56803849	6.66291049	-1.61756044
1	-0.54610940	6.99550905	-1.41901934
1	-2.15516990	7.55266173	-1.87563084
6	-2.20322385	5.90555818	-0.45947531
1	-1.52937007	5.74568888	0.37843995
1	-3.08966657	6.43294342	-0.08528319
8	-2.55380153	4.67470310	-1.01700368
6	-2.63844630	4.88703310	-2.38077778
1	-3.58565263	5.38952432	-2.62444153
1	-2.56986080	3.95709223	-2.93493611
1	6.80246599	0.36923548	-3.59155709
1	8.47173388	0.14271136	-3.06693310
6	7.49635529	0.54199271	-2.76485835
6	7.58978096	2.00758842	-2.36051750

1	8.63548983	2.32934251	-2.29439351
1	7.07789443	2.68769181	-3.04656539
8	6.97387230	2.04648733	-1.10323890
6	7.18110607	0.78614819	-0.56093812
1	8.20666503	0.70963452	-0.16346484
1	6.46749456	0.58131884	0.23851012
8	7.01714502	-0.10220303	-1.61501941

$Li^{+}BF_4^{-}(DD)_{12}$

1	4.08970019	-0.99024681	2.55333282
6	4.27438702	-0.96434286	3.62773503
1	5.19692451	-1.51856063	3.85316999
8	3.21211575	-1.51603370	4.34980511
8	4.36639727	0.33717381	4.09008637
6	4.20224386	0.24025914	5.47642939
1	5.17045243	0.11320516	5.97406158
1	3.73815269	1.14725464	5.85793908
6	3.33325195	-0.99389278	5.64981195
1	3.79730094	-1.72656758	6.31998799
1	2.34530120	-0.75733066	6.04050782
1	-0.13926622	1.54408487	6.36205660
6	0.12924011	1.82799988	5.33180005
1	-0.02861909	2.90675661	5.21858811
8	-0.63506785	1.12742433	4.41708395
8	1.45026405	1.50668732	5.05862034
6	1.51474365	1.12328698	3.70356382
1	2.19781887	1.74370688	3.15415562
1	1.87152788	0.10699871	3.66376583

6	0.10499988	1.23491845	3.22100006
1	-0.07519724	2.19722798	2.75935028
1	-0.18242306	0.46014729	2.52609267
1	-0.73666188	3.05220780	-2.83107734
6	-1.42390476	2.41817894	-2.29685539
1	-1.34570291	2.60543882	-1.23061000
8	-1.20905367	1.08200105	-2.55612289
8	-2.74187362	2.62675925	-2.70637095
6	-3.42286237	1.52736495	-2.19212661
1	-3.81843397	1.74493804	-1.19203566
1	-4.27098187	1.27516464	-2.82868959
6	-2.36399901	0.44212928	-2.13392123
1	-2.24634750	0.06713528	-1.11554447
1	-2.57580996	-0.39790937	-2.78457453
1	0.10645100	4.53588971	-0.07500669
6	0.74088216	5.16184713	-0.71252854
1	0.59875168	6.21522548	-0.41010960
8	0.39871297	4.96712614	-2.03567357
8	2.09118128	4.84747529	-0.57496743
6	2.59038371	4.50008959	-1.83634417
1	3.59595877	4.91391780	-1.96440003
1	2.63837588	3.42416887	-1.93843824
6	1.57616663	5.08725382	-2.76938728
1	1.79595259	6.14154142	-2.96603701
1	1.48061422	4.56937683	-3.71484625
1	-5.35619103	-6.63836596	-0.41006947
6	-5.23342480	-6.14698303	-1.37800879
1	-5.04227718	-6.91249973	-2.13687704
1	-6.17129823	-5.64505718	-1.63825949

8	-4.16049585	-5.23554963	-1.27231645
6	-3.89096672	-4.53682137	-2.45614454
1	-3.65607252	-5.23531353	-3.26434732
1	-4.76470874	-3.95732628	-2.78475755
6	-2.73618394	-3.60265541	-2.23120263
8	-1.58256578	-4.27257533	-1.80266317
6	-0.95702245	-5.06824145	-2.78165198
1	0.02046738	-5.36682093	-2.39737412
1	-0.81520539	-4.52661463	-3.71888447
1	-1.52944455	-5.97485505	-2.99549345
1	-2.99660335	-2.90128858	-1.43806511
1	-2.54868619	-3.02492111	-3.14264242
1	-1.68039883	5.87816730	-3.82693767
6	-1.82172752	5.07141712	-4.54557105
1	-2.60317995	4.40738069	-4.18167217
1	-2.14808977	5.50296106	-5.49924672
8	-0.59470859	4.40091939	-4.67700825
6	-0.60024784	3.30472829	-5.53211408
1	-1.36628639	2.59175798	-5.24213605
1	-0.79833509	3.60882865	-6.57023978
6	0.74013012	2.64671307	-5.43631735
8	0.88226578	2.15419490	-4.14808958
6	2.08642756	1.48424901	-3.87848549
1	2.07221657	1.18733190	-2.83962137
1	2.95180380	2.12593048	-4.05757723
1	2.20156163	0.60000674	-4.51304749
1	1.51745477	3.38166223	-5.66875246
1	0.83020318	1.85292348	-6.19055362
1	-4.26418679	2.31738403	-4.88020343

6	-3.91972149	1.56139657	-5.58338611
1	-4.74444592	0.86983771	-5.79319401
1	-3.66191324	2.05553182	-6.52513667
8	-2.81914529	0.90529272	-5.01355154
6	-2.16313925	0.02388519	-5.86930986
1	-2.78388835	-0.85139731	-6.09003387
1	-1.94440442	0.50584190	-6.83174562
6	-0.88473524	-0.41981550	-5.23633697
8	-1.13262324	-1.38746185	-4.25719992
6	-0.00177297	-1.60740287	-3.44331648
1	-0.26027847	-2.37547605	-2.72366568
1	0.29324106	-0.70511961	-2.92147641
1	0.84762814	-1.96404736	-4.03270002
1	-0.40471270	0.44482894	-4.79693809
1	-0.21919027	-0.83262698	-6.00750894
1	-2.17885818	0.05199147	1.05536399
6	-2.74207670	0.07403940	1.97867649
1	-2.51812725	0.99853758	2.50258577
1	-3.81402551	0.05468128	1.76291490
8	-2.37440194	-1.05096432	2.75054893
6	-2.81801552	-0.99994787	4.06907085
1	-2.35929645	-0.15900160	4.58227989
1	-3.90660402	-0.87217200	4.13176882
6	-2.41405894	-2.29357252	4.70732786
8	-1.08997841	-2.57944291	4.37382948
6	-0.11754270	-1.99205016	5.21144337
1	0.85944167	-2.29068902	4.84661838
1	-0.23308316	-2.34025824	6.24006694
1	-0.18542224	-0.91152440	5.19276897

1	-3.01738941	-3.11104990	4.30930041
1	-2.57743873	-2.26177753	5.79028705
1	3.44543864	-0.58811373	-1.17203498
6	4.10753021	0.14433871	-0.71226215
1	5.14077521	-0.06052585	-1.04082941
8	4.03733790	0.09082353	0.66545947
8	3.75165938	1.43089155	-1.05227491
6	4.22818379	2.26228365	-0.03339710
1	3.45802801	2.96556365	0.24404744
1	5.10157375	2.82995598	-0.37329120
6	4.58947511	1.30807282	1.07834321
1	4.19602654	1.59521237	2.04222267
1	5.67654872	1.21075189	1.16848481
1	1.47185712	3.57307039	1.57064020
6	1.98387458	4.12779597	2.35114716
1	2.13698660	5.16289311	2.02642246
8	3.20517653	3.54056013	2.64855869
8	1.28255117	4.09627230	3.55089044
6	2.22892379	4.46350047	4.50924252
1	2.29292901	5.55457568	4.59094016
1	1.95428021	4.07037109	5.48728025
6	3.52328050	3.89019852	3.96415631
1	4.34100371	4.61949260	3.99592440
1	3.85104180	2.99872198	4.49836557
1	1.75430864	-1.70622441	2.49290182
6	1.45803938	-1.56507601	1.45508499
1	1.75331268	-0.58354848	1.11341096
8	0.08315390	-1.72039785	1.31253578
8	1.98416257	-2.55633818	0.65986310

6	1.20940336	-2.54552535	-0.50310303
1	1.66846636	-1.93161746	-1.27040575
1	1.12726630	-3.56399222	-0.88561112
6	-0.11104251	-1.95893834	-0.05229544
1	-0.32237537	-1.03314692	-0.56229662
1	-0.94333589	-2.63633474	-0.17690612
1	-3.92922587	-2.23766466	1.28619874
6	-3.79139966	-3.31093761	1.34216334
1	-4.31196779	-3.68747009	2.22528818
1	-4.22221474	-3.78422788	0.46521881
8	-2.40860497	-3.57193205	1.42469378
6	-2.09011570	-4.93082740	1.34037850
1	-2.49566870	-5.44949819	2.21413248
1	-2.51923837	-5.38621259	0.44614713
6	-0.60328040	-5.05629685	1.33195744
8	-0.11276678	-4.29950953	2.38939269
6	1.19774974	-4.63309786	2.78618482
1	1.50160948	-3.94099455	3.56653330
1	1.89657695	-4.55622041	1.95785243
1	1.23226337	-5.65050164	3.18646550
1	-0.20086848	-4.69546508	0.38926043
1	-0.32704532	-6.11435783	1.41719979
3	-1.11188474	-2.58470707	2.45407992
9	0.95208420	2.41810357	-0.99703104
5	0.91147249	1.29908386	-0.21201450
9	1.71236222	1.47381068	0.90675945
9	1.35278427	0.19652631	-0.92311633
9	-0.38875519	1.04861856	0.22474341

LiTFSI$(DD)_{12}$

6	-0.64721734	-3.85588408	-2.00925933
6	1.20753882	-3.47051993	-0.96112085
6	0.24913844	-3.08896212	-2.94449056
8	1.35912569	-2.76285647	-2.15075520
1	-1.69202990	-3.57480422	-2.07659839
1	-0.56303336	-4.93302370	-2.17855566
1	-0.22315822	-2.19124756	-3.30412622
1	0.55993124	-3.68833013	-3.80493979
8	-0.13426886	-3.51642616	-0.76752942
6	2.67204077	0.66579275	-5.71743966
6	2.79174824	-1.37031120	-4.87562934
6	3.00500698	0.75353845	-4.24031579
8	2.76713203	-0.54217274	-3.76142608
1	1.91139339	1.37838032	-6.03002893
1	3.56342831	0.81750489	-6.33640288
1	2.37829252	1.45333615	-3.71261051
1	4.05223901	1.02547380	-4.07835253
8	2.18473504	-0.64085728	-5.86789920
6	0.75653421	-1.12219781	3.11275609
6	2.50803403	-1.95579387	2.11812769
6	0.44842233	-2.52963945	2.67215558
8	1.51539079	-2.85666288	1.84434698
1	-0.04473125	-0.41573020	2.96876832
1	1.03658721	-1.10772310	4.16874084
1	-0.47045097	-2.62052652	2.12525769
1	0.40682295	-3.20466501	3.52352976
8	1.84667643	-0.77247013	2.32441516

6	-1.21338228	-5.10348999	1.41195370
8	-1.51829386	-4.88549052	2.74460981
6	-0.67123749	-5.74123104	3.44895324
1	-0.47775455	-5.34226432	4.44815937
6	0.56066184	-5.81274915	2.56699956
8	0.17226739	-5.16793641	1.38802053
1	0.85283393	-6.85082542	2.37209111
1	1.41968579	-5.30152548	2.99349671
1	-1.12702091	-6.73020770	3.57224204
1	-1.63713746	-6.06189304	1.08122504
1	-1.58968695	-4.29442702	0.80106522
1	3.83512178	-1.60318444	-5.13476886
1	2.25884756	-2.30393401	-4.67911558
1	3.04824380	-2.24887874	3.02305821
1	3.20244996	-1.86257960	1.29665730
1	1.71015235	-2.97699727	-0.15323535
1	1.61125142	-4.47566542	-1.10457827
6	-1.94402260	6.15452324	-1.05091281
1	-0.88467362	6.01237335	-1.24334696
1	-2.06720498	6.95966681	-0.32442159
1	-2.42820554	6.46049277	-1.98597744
1	-2.27714639	4.10438600	-2.35782360
6	-2.33718353	3.84985576	-1.29957585
1	-1.39902267	3.37117387	-1.04073918
8	-2.54540112	4.99581492	-0.51937326
6	-3.47788208	2.89771581	-1.10284687
1	-3.59616535	2.69201684	-0.04292102
8	-3.24759002	1.72447312	-1.82369701
1	-4.39785281	3.38885989	-1.43368440

6	-4.38225028	0.91741376	-1.96168820
1	-5.04483151	1.28677987	-2.75404971
1	-4.97027230	0.86546569	-1.04799222
1	-4.05625413	-0.08654147	-2.21946832
6	0.58252027	6.69358861	1.77852737
1	0.95183145	6.26464460	2.71463981
1	-0.41786947	7.09279424	1.95186343
1	1.24932166	7.51899335	1.50396714
1	2.38587095	5.80371553	-0.03953079
6	1.69555480	5.10055039	0.43954824
1	2.20311132	4.72422268	1.33777093
8	0.49451059	5.73042446	0.75654175
6	1.41528624	3.94564351	-0.47512625
1	2.33084056	3.36753708	-0.61375363
8	0.88407424	4.35311362	-1.70576038
1	0.68631439	3.29916541	-0.00193596
6	1.84357714	4.74269315	-2.64820644
1	2.50668441	3.91636643	-2.92177707
1	2.47202200	5.56325568	-2.29013091
1	1.32696177	5.08209793	-3.54823223
6	4.51230403	-3.10109355	-2.30629999
1	4.85211181	-2.89901948	-3.32474776
1	3.62870146	-3.72394072	-2.34328085
1	5.30288108	-3.64260405	-1.79234740
1	6.14097510	-1.54890367	-1.15587957
6	5.26491905	-1.02743065	-1.54591183
1	5.53322103	-0.64856549	-2.53928956
8	4.18606389	-1.90826430	-1.63624270
6	4.93826938	0.10619467	-0.63594441

1	5.74729780	0.84911625	-0.69615426
8	4.80283268	-0.37887932	0.65783932
1	4.02309596	0.58093676	-0.96643701
6	4.44932596	0.61255692	1.59866550
1	3.45870859	1.00716819	1.40145005
1	5.17146435	1.43455555	1.59252494
1	4.45525133	0.15598213	2.58592374
3	2.42080837	-1.24545569	-2.03594357
1	-3.27295205	-2.05584729	-2.91915987
6	-3.26026713	-2.12862166	-4.00555151
1	-4.14702197	-1.61907396	-4.39465649
1	-3.31878076	-3.18066123	-4.29465091
8	-2.07341176	-1.59534286	-4.54237388
6	-1.88095727	-0.23740026	-4.23940568
6	-0.62424913	0.22357744	-4.91964485
1	-0.83574673	0.48957185	-5.95830118
1	-1.78598839	-0.10540359	-3.17644269
1	0.07125332	-0.60413496	-4.93816384
1	-2.73878452	0.35263446	-4.56782898
8	0.01462444	1.27699289	-4.26313013
6	-0.70829440	2.47719350	-4.26829624
1	-1.68902079	2.35882726	-3.82052564
1	-0.84305990	2.85845743	-5.28509229
1	-0.15105926	3.20742104	-3.69037064
1	-3.09582134	5.69120049	1.97907389
6	-3.36255281	4.92761036	2.71175722
1	-4.03161567	4.22144361	2.22901461
1	-3.89505654	5.40426274	3.53848050
8	-2.22446537	4.29312433	3.24167685

6	-1.42704873	3.68148088	2.26962264
6	-0.16471078	3.20012399	2.89468760
1	0.29033726	4.02915330	3.44045663
1	-1.18478073	4.37837142	1.47113971
1	0.53165212	2.87917002	2.12262394
1	-1.94609223	2.84740749	1.83231811
8	-0.41820050	2.13680387	3.76859769
6	0.70664604	1.79552267	4.53664987
1	1.06920383	2.64759107	5.12194090
1	1.52959124	1.43807039	3.91417321
1	0.42272446	1.00499231	5.23358074
8	3.70972829	-4.72384280	2.44765364
6	5.02007063	-4.23226132	2.42174432
1	5.15394250	-3.49857953	3.22001128
1	5.74687945	-5.03577491	2.58778895
6	5.16079648	-3.64669643	1.02279589
1	5.34067388	-2.57503779	1.00996465
1	5.97699892	-4.13441932	0.47943889
8	3.92857827	-3.91813370	0.41962567
6	3.39591880	-4.98521830	1.12743315
1	3.87490311	-5.92198740	0.80279654
1	2.32229409	-5.05478942	0.98416347
8	-4.22603651	-3.62279093	1.84772025
6	-4.06789134	-3.54156456	3.23231240
1	-5.04139998	-3.52785453	3.73227818
1	-3.49027261	-4.38127231	3.60428251
6	-3.40539775	-2.20835167	3.35023531
1	-3.49468510	-1.73949097	4.33352586
1	-2.35791858	-2.29083313	3.08708792

8	-4.09206916	-1.46380148	2.39636227
6	-4.60236692	-2.34929449	1.45391003
1	-4.22211751	-2.16073998	0.45251502
1	-5.69972025	-2.23952714	1.44326556
8	-5.61149150	2.21505420	3.28278421
6	-4.65383473	2.12518777	4.31075882
1	-4.42468424	3.12656781	4.67878442
1	-5.03585920	1.53559811	5.15210106
6	-3.46780801	1.48408982	3.63976462
1	-2.50970801	1.86831363	3.95966928
1	-3.49181290	0.40883198	3.78007268
8	-3.69322635	1.77740455	2.29811225
6	-5.05109012	1.62101101	2.15579980
1	-5.31178419	0.55970899	2.11962564
1	-5.41077751	2.10923967	1.24175449
9	0.61567552	-0.36062728	-2.39045858
6	0.42464608	0.68104375	-1.64342049
9	-0.74468020	1.14950821	-1.75735527
16	0.93007749	0.10689235	-0.00596273
9	1.30654144	1.58657350	-1.96461215
7	0.15391002	-1.19230421	0.30828096
8	2.25636294	-0.35672755	-0.39873322
8	0.82053959	1.20503939	0.92240648
16	-1.36105131	-1.24740018	-0.06074091
8	-1.93943532	-2.32614686	0.70267543
8	-1.63001074	-1.20267099	-1.48264339
9	-1.65991890	1.31446070	0.39846359
6	-2.10930806	0.17451430	0.76222311
9	-1.84683485	-0.01221255	2.01072542

9	-3.36665577	0.08122034	0.49555064

$Li^+TFSI^-(DD)_{12}$

1	-2.48662891	-5.32834105	-2.20359616
6	-1.64569680	-5.64792905	-1.57896327
1	-1.56031653	-6.74580083	-1.64272192
8	-1.82993782	-5.25783399	-0.26959409
8	-0.46460440	-5.05181731	-1.97858540
6	0.38563729	-5.14296525	-0.86967004
1	0.99529027	-6.05058185	-0.93256472
1	1.04230851	-4.28438639	-0.83910638
6	-0.55751853	-5.19668947	0.31140375
1	-0.37612349	-6.08103953	0.93177088
1	-0.49737311	-4.31623277	0.93821366
1	3.37760239	3.25833550	0.57528005
6	3.15046002	2.47177616	-0.15921085
1	3.28549532	1.50051934	0.29462888
8	3.94820715	2.60909823	-1.26869547
8	1.83478675	2.57995074	-0.60486110
6	1.82671450	2.26199046	-1.97369656
1	1.14883082	1.45053752	-2.19150479
1	1.50368081	3.14845463	-2.52547498
6	3.25768483	1.92186437	-2.27628967
1	3.41368439	0.85756218	-2.19063501
1	3.60115670	2.27311445	-3.25150109
1	-6.66539605	1.67776795	2.10005740
6	-5.75179432	1.15028225	1.77556845
1	-5.95052781	0.66335718	0.81303063

8	-5.35869777	0.19793148	2.69610559
8	-4.71578182	2.08696850	1.68151786
6	-3.70189029	1.70912086	2.57820072
1	-2.93278955	1.15732311	2.05779525
1	-3.25966383	2.59221563	3.02789255
6	-4.43828506	0.82601163	3.54027639
1	-3.81725453	0.08051157	4.04076779
1	-4.95237281	1.41654116	4.30578161
1	3.25329800	-4.73951000	4.70336713
6	3.15857438	-3.77989854	4.16721556
1	3.83883357	-3.05768040	4.61760914
8	1.85649012	-3.33303567	4.21668032
8	3.44299824	-3.96432811	2.83076358
6	2.25983739	-4.37537881	2.21589290
1	2.11987191	-3.83723477	1.28818362
1	2.29896755	-5.44433706	1.98000221
6	1.18754419	-4.07407215	3.23796332
1	0.35905975	-3.50183722	2.83790248
1	0.79120702	-4.99938568	3.66942365
1	-2.62860590	-3.10800840	-2.82957845
6	-2.14893456	-2.83114432	-3.75358337
1	-1.32861686	-3.51946997	-3.94850672
1	-2.87099082	-2.92590068	-4.56958044
8	-1.68753923	-1.50906978	-3.62934185
6	-1.24089036	-0.97913018	-4.83341493
1	-0.51251511	-1.64134001	-5.30711513
1	-2.07615152	-0.87446273	-5.54164110
6	-0.60726382	0.35659060	-4.60175239
8	0.61891427	0.23713147	-3.95358569

6	1.72737844	0.23673673	-4.81757621
1	2.61247535	0.02950837	-4.22908022
1	1.84044820	1.20268878	-5.32145800
1	1.63995926	-0.52673231	-5.59373976
1	-1.26100079	0.94284605	-3.97055142
1	-0.50123586	0.87656578	-5.56114161
1	-5.57061312	3.53189745	0.27745055
6	-5.65409901	4.17538452	-0.58804538
1	-6.04216054	3.59669196	-1.42791290
1	-6.35960604	4.98474078	-0.38385237
8	-4.37047124	4.69111682	-0.88092116
6	-4.32210422	5.37740884	-2.09779713
1	-4.59689225	4.70828733	-2.91767988
1	-5.02593303	6.21960647	-2.11693080
6	-2.91373259	5.84648789	-2.27734512
8	-2.07841482	4.74587774	-2.11040473
6	-1.46146916	4.26312532	-3.28380407
1	-0.89613707	3.37616836	-3.02815120
1	-0.79344948	5.01716594	-3.70721980
1	-2.20366483	4.00637013	-4.04147970
1	-2.67040415	6.58218554	-1.50685612
1	-2.79183062	6.33936570	-3.24741223
1	3.48522434	-0.19202197	4.16424297
6	4.55505565	-0.33377230	4.10978755
1	5.00681313	0.57044102	3.69877437
1	4.95305860	-0.46854025	5.12058737
8	4.81007607	-1.45126079	3.30592744
6	6.15750027	-1.74713324	3.16288630
1	6.66138598	-0.99602176	2.54982821

1	6.65744795	-1.74635207	4.14123807
6	6.32757201	-3.09085899	2.53148346
8	6.06594000	-3.02280803	1.16605872
6	5.71983518	-4.25837697	0.60020837
1	5.57621835	-4.11527415	-0.46923819
1	4.80196007	-4.64773541	1.03198144
1	6.50850876	-5.00515800	0.74126154
1	5.65127892	-3.78205364	3.02215699
1	7.35468051	-3.44689342	2.70361059
1	5.47169732	0.89242697	0.36828440
6	5.83576907	0.09267256	-0.26867036
1	6.56166661	0.51984516	-0.96723283
1	6.34918009	-0.64337728	0.34807126
8	4.74436085	-0.47088760	-0.93210843
6	5.08852597	-1.49610809	-1.79725665
1	5.46422441	-1.08680235	-2.74182969
1	5.88889147	-2.10821268	-1.37139138
6	3.92331889	-2.38357146	-2.08870704
8	2.96227056	-1.71570490	-2.83452119
6	1.88611472	-2.54147799	-3.20296894
1	1.07546166	-1.91374630	-3.53577275
1	1.53311424	-3.13198091	-2.37163024
1	2.17045315	-3.22452546	-4.01037796
1	3.50096158	-2.73951748	-1.15867728
1	4.29272908	-3.26482008	-2.63270069
1	-4.67353409	-1.74491442	-2.23348348
6	-4.78686472	-2.20379067	-1.24325290
1	-5.76271273	-2.71815169	-1.21378652
8	-4.72494163	-1.25052288	-0.24458926

8	-3.76799963	-3.09232455	-0.99116975
6	-3.69758308	-3.21243232	0.39790328
1	-2.67140244	-3.33488992	0.70605245
1	-4.25357501	-4.09006436	0.73403387
6	-4.32285363	-1.93457133	0.91212573
1	-3.62751653	-1.31456553	1.45673864
1	-5.18378514	-2.14068733	1.55011233
1	1.36603861	2.58929616	3.17561084
6	1.06566670	1.67379019	2.63700863
1	1.43399953	1.73628961	1.61565829
8	-0.31169086	1.54604309	2.62216562
8	1.56999333	0.58558277	3.31273440
6	0.52252963	-0.16264904	3.83318228
1	0.35295308	-1.05101091	3.24751660
1	0.74963215	-0.47509901	4.85491566
6	-0.63935556	0.77313061	3.73107440
1	-1.58968250	0.27494319	3.57137523
1	-0.71591307	1.39943945	4.62584722
1	-0.40843726	2.70097196	-0.68891816
6	-1.14991162	1.91474848	-0.66371437
1	-0.80012072	1.13256697	-0.01497891
8	-1.40035572	1.42783174	-1.92937831
8	-2.35813437	2.41591657	-0.20591990
6	-3.30857666	1.54268714	-0.73642602
1	-3.31792970	0.62794284	-0.17475548
1	-4.28901380	1.99427463	-0.71075555
6	-2.77367775	1.33092562	-2.11016109
1	-3.04945229	0.38333856	-2.55831099
1	-3.10806435	2.12692217	-2.77994300

1	-4.95800165	4.64258873	1.88486253
6	-4.09784351	5.11991464	2.33412181
1	-4.17966816	6.20399537	2.21832310
1	-4.09203161	4.89511012	3.40168432
8	-2.93608039	4.63002051	1.70456836
6	-1.76150165	5.01211794	2.36056110
1	-1.70546220	6.10335967	2.41358634
1	-1.73419007	4.63411541	3.38938466
6	-0.61331605	4.48019294	1.57443117
8	-0.80190993	4.89389468	0.25429245
6	0.35201384	5.40221093	-0.38296593
1	0.06985597	5.71478224	-1.38252794
1	1.12686271	4.64520452	-0.44664329
1	0.73879750	6.26850253	0.15911265
1	-0.60007527	3.39716094	1.62273257
1	0.32683379	4.84516678	1.99573325
3	-2.57461670	4.25170420	-0.20608168
9	-2.29290270	-1.05713544	-1.12077006
6	-1.11293378	-1.49710114	-0.86219145
9	-0.24572131	-0.84091040	-1.54468369
16	-0.83713935	-1.13291754	0.89919985
8	-1.91031620	-0.21371166	1.22605044
7	0.48050739	-0.29533964	0.86866232
8	-0.80311879	-2.37432791	1.65273933
9	-1.05439040	-2.75323898	-1.09125516
8	1.34066203	-2.39851852	-0.28864955
16	1.64519614	-1.03174205	0.09788932
8	2.23171119	-0.11882894	-0.83082125
6	2.88646436	-1.23200083	1.37558193

9	2.31692819	-1.67193947	2.43515122
9	3.41998382	-0.06883114	1.58431184
9	3.76636224	-2.06446758	0.95917764

$\underline{Li^+(DME)_4}$

3	0.08100722	-0.42347802	0.32973343
1	-1.33637292	1.22748276	3.65281730
1	-0.33844122	1.98331579	2.40532541
6	-1.22347832	1.38235641	2.57752659
8	-1.06721915	0.15669789	1.90530716
1	-2.09741728	1.92028268	2.21859397
6	-2.08147993	-0.75676988	2.18054141
1	-2.93650493	-0.56337102	1.53565217
1	-2.43440431	-0.67980385	3.21521483
6	-1.53319097	-2.12248929	1.94271198
1	-2.33484490	-2.86616862	2.02288792
1	-0.79112901	-2.36791219	2.70470289
8	-0.93621512	-2.12678545	0.68448218
1	-0.38390356	-3.30347556	-0.87333673
1	-0.24008104	-4.07890272	0.71118360
6	-0.84475635	-3.40601302	0.10088979
1	-1.83343397	-3.85596870	-0.01566901
1	0.26863188	3.02063463	-1.05654240
1	-1.01857442	2.26786443	-0.10562195
6	0.01832757	2.57342923	-0.09593677
8	0.81241192	1.44054763	0.16795060
1	0.16222480	3.34132884	0.66516922
6	2.16548378	1.73848736	0.36921002

1	2.61324418	0.86299929	0.82126178
1	2.28148447	2.57606113	1.06705153
6	2.88211094	2.06265052	-0.90695544
1	3.93229002	2.28457963	-0.67335011
1	2.46470165	2.96867940	-1.35576568
8	2.76845182	0.98368517	-1.78728277
1	3.37505856	0.26938411	-3.59882181
1	3.22022893	2.03657461	-3.52872919
6	3.53036366	1.14823907	-2.97153430
1	4.59774507	1.24020456	-2.75356389
1	1.65029581	-2.91372543	-2.32557594
1	0.31271966	-1.78222037	-2.55749750
6	1.32415143	-1.88131666	-2.18181246
8	1.32813964	-1.53675508	-0.81800981
1	1.98401599	-1.23385460	-2.75380221
6	2.54857095	-1.77592521	-0.19172787
1	3.21800964	-0.93343923	-0.35511536
1	3.04792999	-2.66617227	-0.59053577
6	2.27774359	-1.96662005	1.26168178
1	3.22321766	-2.03211768	1.81294414
1	1.74602828	-2.90580050	1.42492599
8	1.49187172	-0.89950218	1.68966028
1	0.95569016	0.20948619	3.30201495
1	1.24907348	-1.49920367	3.65887687
6	1.58333264	-0.64210953	3.07196860
1	2.61238208	-0.41748894	3.36211933
1	-1.11646633	1.58885163	-2.55460480
1	0.45235897	0.81082524	-2.30748820
6	-0.60845850	0.63546862	-2.41958618

8	-1.07084190	-0.03757321	-1.27205325
1	-0.78432202	0.04541934	-3.31996418
6	-2.40065546	-0.46463004	-1.37033666
1	-2.55720554	-1.17943679	-0.57306956
1	-2.57682668	-0.97634935	-2.32375409
6	-3.38130328	0.66061474	-1.23032864
1	-4.39781161	0.25319878	-1.31629539
1	-3.26101396	1.37263001	-2.05198366
8	-3.18379900	1.29424050	-0.00085183
1	-3.92152170	2.72952240	1.24625367
1	-4.16338580	3.07104232	-0.47909060
6	-4.15981290	2.28357898	0.27962677
1	-5.16442610	1.85501521	0.32830782

$Li^+(DME)_3$

3	0.00547735	0.00094734	-0.00598821
1	0.09474827	1.56975353	3.57372897
1	0.74708972	2.23009997	2.05961721
6	-0.12601162	1.78492356	2.52555597
8	-0.44243082	0.60390247	1.82224900
1	-0.95104270	2.49909562	2.49545670
6	-1.58688399	-0.04632875	2.27833888
1	-2.47709965	0.45322565	1.89041127
1	-1.66222626	-0.03335813	3.37237865
6	-1.53929168	-1.45779783	1.79261923
1	-2.48440213	-1.96220428	2.02742982
1	-0.74753242	-2.00619263	2.30719568
8	-1.28415761	-1.43137873	0.42340227

1	-1.34830192	-2.50214371	-1.29828799
1	-1.08430108	-3.47907653	0.15828613
6	-1.61937095	-2.62174601	-0.25439944
1	-2.69001617	-2.82701474	-0.18169683
1	-3.16753552	1.29266219	-1.91040752
1	-2.99900017	-0.14472503	-0.88158795
6	-2.79746161	0.91914511	-0.95269898
8	-1.40471763	1.10905647	-0.83814263
1	-3.33663217	1.43875798	-0.15844639
6	-1.00228311	2.44228329	-0.87574684
1	-1.17993746	2.91035333	0.09461513
1	-1.56168395	3.02241194	-1.61963686
6	0.45436916	2.47458350	-1.20357580
1	0.83097071	3.50114669	-1.11767194
1	0.61321573	2.16128836	-2.23774231
8	1.10882584	1.60122039	-0.33786749
1	2.91033928	1.05414526	0.41780104
1	2.97596247	1.68739740	-1.23757185
6	2.50330414	1.79755102	-0.25976587
1	2.73909854	2.79557585	0.11706026
1	-0.04580124	-1.50084356	-3.61912907
1	-0.94610551	-0.11242056	-2.97536085
6	0.03184789	-0.57826569	-3.03907719
8	0.47702182	-0.84032080	-1.72666135
1	0.71435207	0.09267275	-3.56407472
6	1.74336347	-1.41617669	-1.65469043
1	2.50789181	-0.65482158	-1.82410222
1	1.88883823	-2.19201013	-2.41626326
6	1.90302660	-2.00940221	-0.29341612

1	2.93385177	-2.36002146	-0.16149363
1	1.25342131	-2.88032255	-0.18439085
8	1.55397692	-1.03832334	0.64246932
1	1.67831999	-0.49347415	2.59275626
1	1.66373499	-2.24668792	2.32516916
6	2.02956025	-1.29076321	1.94591010
1	3.12161908	-1.31637945	1.96744354

$Li^+(DOL)(DME)_3$

3	0.00000000	0.00000000	0.00000000
1	0.00000000	0.00000000	4.28921682
1	1.34971151	0.00000000	3.10554617
6	0.35612737	0.40626006	3.32714498
8	-0.50909741	0.00224195	2.26011010
1	0.41439686	1.50544534	3.39480258
6	-1.85001910	0.46468971	2.47011843
1	-1.88863153	1.56049814	2.33208269
1	-2.18390028	0.22533358	3.49654568
6	-2.75808365	-0.24517314	1.48851432
1	-3.78867754	0.13758614	1.60692543
1	-2.76518917	-1.33400686	1.68461598
8	-2.28244693	-0.00002418	0.15982785
1	-2.73153935	-0.34308931	-1.80460861
1	-3.32429084	-1.61189798	-0.68663570
6	-3.18267167	-0.52555232	-0.82408968
1	-4.16239891	-0.02286418	-0.76037854
1	3.28863192	2.19555983	0.40016722
1	1.72151202	2.24594433	1.26629365

6	2.61348393	1.63783764	1.06899670
8	2.16199482	0.41541461	0.47050201
1	3.14324554	1.43446993	2.01625739
6	3.22584944	-0.52265585	0.23523962
1	2.72689480	-1.47707094	0.01946261
1	3.82838860	-0.64153116	1.15558245
6	4.15964768	-0.15725087	-0.91072294
1	4.95789619	-0.92617724	-0.94840099
1	4.65896827	0.81652825	-0.73788853
8	3.43211088	-0.12806729	-2.13496430
1	3.66282951	0.13777036	-4.14854253
1	4.78972523	1.11257222	-3.14881819
6	4.28960102	0.13224850	-3.24782186
1	5.06157259	-0.65114601	-3.35045995
1	-0.57918751	-0.69013308	-4.19453779
1	-0.66020178	0.81608921	-3.22124448
6	-0.11693121	-0.12455648	-3.36754760
8	-0.21263024	-0.85864425	-2.14183348
1	0.93491852	0.09270336	-3.61765973
6	0.44751037	-2.12784416	-2.23910688
1	1.54124706	-1.97347924	-2.27464954
1	0.13443715	-2.65543370	-3.15886380
6	0.04934170	-2.96486803	-1.04221420
1	0.58692865	-3.93037709	-1.08085141
1	-1.03783198	-3.16960069	-1.05694038
8	0.38733175	-2.25117124	0.15285050
1	0.40346829	-2.42926908	2.18940078
1	-0.89098448	-3.36997586	1.38295563
6	0.16435139	-3.05137919	1.32105633

1	0.81127503	-3.94464194	1.30788277
8	-0.04194514	1.92750669	-0.75356389
6	0.75477501	2.36674589	-1.88196529
8	-0.15649404	2.56989678	-2.92696242
1	1.45948662	1.57006544	-2.14716838
1	1.27399547	3.30506392	-1.60695306
6	-1.22379047	3.28979193	-2.28610692
1	-2.12742794	3.17919228	-2.89577384
1	-0.96304902	4.35811073	-2.19867943
6	-1.32072071	2.61346521	-0.89658536
1	-2.11496107	1.85554774	-0.85241049
1	-1.44829960	3.33819550	-0.08042130

$Li^+(DOL)_2(DME)_2$

1	0.93199739	3.37566051	1.13694464
6	0.23473140	2.97238396	0.38879651
1	-0.73019378	2.80166957	0.86138147
8	0.75561100	1.78758549	-0.13271737
8	0.12164531	3.80955973	-0.69542276
6	1.32845079	3.63948732	-1.39121835
1	1.18406785	3.86398728	-2.45037775
1	2.10138133	4.31137889	-1.00554081
6	1.68033441	2.18402110	-1.11118233
1	1.57258633	1.54562457	-1.98168664
1	2.70438534	2.08083516	-0.74338662
3	-0.06738601	-0.01457927	0.01920722
1	-2.06255035	-3.15259739	-0.68206009
6	-1.00482306	-2.86390208	-0.60963408

1	-0.64987449	-2.57102703	-1.59586008
8	-0.24585352	-3.87709370	-0.07295075
8	-0.86924985	-1.82390431	0.30534457
6	-0.93849513	-2.43116590	1.56630650
1	-0.23640380	-1.94779536	2.23598234
1	-1.93874976	-2.32764629	1.99211199
6	-0.59981616	-3.89003490	1.28632593
1	0.23071436	-4.27417246	1.88227042
1	-1.46461403	-4.53746309	1.45957601
1	-1.80775744	0.18136640	3.49797010
1	-0.09073859	0.45064205	3.14149091
6	-1.10393504	0.75316708	2.89000937
8	-1.30175867	0.51421023	1.51379401
1	-1.23146734	1.81012143	3.13498028
6	-2.57266791	0.86533672	1.05574701
1	-2.67010898	1.95411632	1.02622067
1	-3.36292320	0.49894748	1.72117435
6	-2.74404859	0.28930178	-0.31330383
1	-3.66168773	0.67375511	-0.77001821
1	-2.83879006	-0.78966160	-0.24702399
8	-1.60714559	0.57861670	-1.07012416
1	-0.83387885	1.79663691	-2.50079920
1	-2.52474138	1.33721199	-2.76389657
6	-1.78423857	1.61666162	-2.01101215
1	-2.10775352	2.53919193	-1.53391177
1	3.21879828	0.55196771	1.17403554
1	1.89383825	0.99286497	2.25742509
6	2.42734447	0.15806998	1.81158628
8	1.50798853	-0.61907636	1.07812942

1	2.89245397	-0.42969970	2.60680262
6	2.08926169	-1.68121669	0.37907111
1	1.43531696	-2.53733094	0.46342293
1	3.06442700	-1.96439732	0.78606112
6	2.21544672	-1.30595096	-1.05901874
1	2.53422542	-2.17608913	-1.64330018
1	2.97775226	-0.53403554	-1.19347791
8	0.97458494	-0.82091795	-1.47672161
1	-0.20062088	-0.43763457	-3.08587744
1	1.53609492	-0.23323917	-3.38768436
6	0.78644896	-0.83914180	-2.87431438
1	0.85238443	-1.85460645	-3.26964627

$Li^+(DOL)_3(DME)$

1	-0.08831039	2.42677319	1.42027608
6	0.43380519	2.66783263	0.49617895
1	-0.11393413	3.45788030	-0.03579846
8	0.53340023	1.54490409	-0.33891138
8	1.73277832	3.03524387	0.73129744
6	2.33294006	2.97614606	-0.53539446
1	3.41588161	2.87558966	-0.43708051
1	2.12582818	3.88406566	-1.10931777
6	1.65416368	1.76866532	-1.16044328
1	2.28408487	0.88455340	-1.14415090
1	1.34747568	1.94767406	-2.19313772
1	-2.78157822	-0.71121898	-2.49361059
6	-1.76881157	-0.50637511	-2.12265658
1	-1.20583333	-1.43653489	-2.09692159

8	-1.83905703	0.07949160	-0.85936685
8	-1.13721472	0.45389111	-2.87930324
6	-1.77369173	1.66009206	-2.52870757
1	-1.04763563	2.47180589	-2.55405877
1	-2.57547007	1.90052230	-3.23377327
6	-2.32170321	1.37251846	-1.13667648
1	-1.98467061	2.06777004	-0.37454087
1	-3.41557806	1.37103040	-1.13290442
3	-0.15731115	-0.27091606	0.00751966
1	2.09570816	-0.31252608	2.93800071
6	1.50657764	0.44279459	2.40006310
1	2.12652412	0.91572710	1.64297170
8	0.95845515	1.36082493	3.25771588
8	0.39190329	-0.18716102	1.82376744
6	-0.55060554	-0.31403450	2.86048222
1	-1.53682748	-0.05116681	2.47874690
1	-0.59476141	-1.34368113	3.22246923
6	-0.04846127	0.64914922	3.92949707
1	-0.81060971	1.33909494	4.29803831
1	0.36030835	0.10490526	4.78592417
1	-2.12228764	-3.34839577	1.50708552
1	-2.82091198	-1.85822106	0.83222364
6	-2.14329977	-2.68688925	0.63788362
8	-0.86151156	-2.15230932	0.37335363
1	-2.51719371	-3.25983417	-0.21348938
6	0.10456366	-3.10428100	0.04868598
1	-0.16777042	-3.61397108	-0.88072651
1	0.20142514	-3.87905772	0.81898288
6	1.39646732	-2.36747625	-0.11994403

1	2.17614645	-3.02929994	-0.50955559
1	1.73188438	-1.99075729	0.84317716
8	1.16839161	-1.25971018	-0.94866627
1	1.14746122	-0.60814513	-2.87979977
1	2.59294831	-1.54214375	-2.42581256
6	1.51077267	-1.45752457	-2.30894886
1	1.05514504	-2.36731506	-2.70064361

$Li^+(DOL)_4$

1	-0.56308777	-1.75871608	-2.29730001
6	-1.56542587	-1.61369040	-1.89530381
1	-2.22143320	-1.20548210	-2.67610374
8	-1.55017091	-0.71273732	-0.80860435
8	-2.06671637	-2.77010224	-1.35720012
6	-3.16989843	-2.34926988	-0.59615425
1	-3.40297362	-3.09134352	0.17028253
1	-4.05375486	-2.21938900	-1.22718034
6	-2.69830370	-1.01341474	-0.03988356
1	-2.41467084	-1.06587758	1.01028751
1	-3.45146284	-0.22771747	-0.15089182
1	1.52459632	-0.47438387	2.35605689
6	0.76210157	0.21689759	2.72747754
1	0.32932172	-0.18546538	3.65343405
8	-0.26280151	0.38679026	1.78051064
8	1.25994395	1.48371647	2.89943836
6	0.11996801	2.29888258	3.02188162
1	-0.22543237	2.32168637	4.05948737
1	0.35651103	3.31491664	2.71012680

6	-0.89235479	1.60323466	2.12481149
1	-1.83507574	1.40858322	2.64457929
1	-1.10695708	2.15999415	1.21930527
1	2.44798912	2.50458460	-0.78844970
6	1.62649826	2.26843750	-0.09903531
1	2.02181512	1.79320267	0.79628422
8	0.71204120	1.42813575	-0.76106386
8	0.88466093	3.38151117	0.20652025
6	0.18239070	3.66732474	-0.97628104
1	-0.72161035	4.23549947	-0.74683892
1	0.79379507	4.26301570	-1.65998823
6	-0.08703313	2.28258573	-1.55171397
1	-1.12833238	1.97464925	-1.46462340
1	0.19908395	2.21171147	-2.60519232
3	0.01168528	-0.12184277	0.00676003
1	1.46674042	-3.42277536	0.60986691
6	0.83984581	-2.88735564	-0.11587333
1	-0.21005872	-2.99745730	0.15321151
8	1.07716495	-3.29946900	-1.40153475
8	1.22231783	-1.52878530	-0.12107808
6	2.34747471	-1.44810554	-0.97348287
1	2.22148631	-0.59856796	-1.64296348
1	3.25992342	-1.29087884	-0.39073745
6	2.34930066	-2.78472696	-1.70201617
1	2.47362172	-2.69939168	-2.78355039
1	3.13727640	-3.43841407	-1.31727700

$\underline{Li^{+}(GBL)_4}$

6	17.71814300	-7.96839827	2.82962109
6	17.41945193	-6.85692651	1.81690510
6	16.75884737	-5.78038348	2.65911366
6	17.84844654	-7.17958272	4.13915299
8	16.99054623	-6.00419973	3.96997586
8	16.11310079	-4.82376323	2.27249450
1	18.62831797	-8.54112076	2.60314322
1	16.87095968	-8.66915308	2.88908755
1	16.76474359	-7.14586555	0.98309189
1	18.87478758	-6.81863528	4.31561090
1	17.49111217	-7.71583239	5.02817094
6	13.95375870	-1.36203020	-1.92569354
6	13.21519403	-1.61424193	-0.60623704
6	14.33578591	-1.92282868	0.37020789
6	15.32324616	-0.87365746	-1.43586282
8	15.52021388	-1.51451816	-0.13330756
8	14.24677694	-2.44816376	1.46439268
1	13.46229432	-0.62344502	-2.57430238
1	14.06329095	-2.30222464	-2.48807783
1	12.48792263	-2.43760877	-0.62581723
1	12.69221606	-0.71338939	-0.23744409
1	15.35150524	0.21692854	-1.27787868
1	16.16507200	-1.17355213	-2.07389153
6	18.04962330	0.95341294	5.00222018
6	18.43331807	-0.37776074	4.34577790
6	17.10687729	-0.91627560	3.84075025
6	16.78397770	1.33895561	4.22532631
8	16.18537238	0.07004074	3.80446102
8	16.84838611	-2.05339634	3.49234689

1	18.82849947	1.72505257	4.92681678
1	17.81205080	0.80038853	6.06634068
1	18.91322759	-1.10765540	5.01225891
1	19.09153472	-0.24114570	3.46880896
1	17.00699021	1.91973135	3.31533688
1	16.03050890	1.86906272	4.82275124
3	15.33423196	-3.15535691	2.92535116
6	11.47072279	-4.87178304	6.23527001
6	12.33312036	-3.61155504	6.09996586
6	13.14774967	-3.87459790	4.84625002
6	11.36741899	-5.34520168	4.77936919
8	12.59555738	-4.87797645	4.13196478
8	14.15211181	-3.29558086	4.47510832
1	10.48194407	-4.68616557	6.67752550
1	11.98974867	-5.62663136	6.84609456
1	12.99646088	-3.40330731	6.95083161
1	11.72839742	-2.70510261	5.91685761
1	10.51696639	-4.89092431	4.24541012
1	11.33371399	-6.43619470	4.65985159
1	18.33875542	-6.42144036	1.38549812
